# Protein-based biorefining driven by nitrogen-responsive transcriptional machinery

**DOI:** 10.1186/s13068-020-1667-5

**Published:** 2020-02-26

**Authors:** Lianjie Ma, Liwei Guo, Yunpeng Yang, Kai Guo, Yajun Yan, Xiaoyan Ma, Yi-Xin Huo

**Affiliations:** 1 Key Laboratory of Molecular Medicine and Biotherapy, School of Life Science, Beijing Institute of Technology, 5 South Zhongguancun Street, Haidian District, Beijing, 100081 People’s Republic of China; 2 Biology Institute, Shandong Province Key Laboratory for Biosensors, Qilu University of Technology (Shandong Academy of Sciences), Jinan, 250103 China; 3 School of Chemical, Materials and Biomedical Engineering, College of Engineering, University of Georgia, Athens, GA 30602 USA

**Keywords:** Amino acid, Higher alcohol, Transcription regulation, Stationary phase, Stress

## Abstract

**Background:**

Protein-based bioconversion has been demonstrated as a sustainable approach to produce higher alcohols and ammonia fertilizers. However, owing to the switchover from transcription mediated by the bacterial RNA polymerase σ^70^ to that mediated by alternative σ factors, the biofuel production driven by σ^70^-dependent promoters declines rapidly once cells enter the stationary phase or encounter stresses. To enhance biofuel production, in this study the growth phase-independent and nitrogen-responsive transcriptional machinery mediated by the σ^54^ is exploited to drive robust protein-to-fuel conversion.

**Results:**

We demonstrated that disrupting the *Escherichia coli* ammonia assimilation pathways driven by glutamate dehydrogenase and glutamine synthetase could sustain the activity of σ^54^-mediated transcription under ammonia-accumulating conditions. In addition, two σ^54^-dependent promoters, *argTp* and *glnAp2*, were identified as suitable candidates for driving pathway expression. Using these promoters, biofuel production from proteins was shown to persist to the stationary phase, with the net production in the stationary phase being 1.7-fold higher than that derived from the optimal reported σ^70^-dependent promoter *P*_L_lacO_1_. Biofuel production reaching levels 1.3- to 3.4-fold higher than those of the σ^70^-dependent promoters was also achieved by *argTp* and *glnAp2* under stressed conditions. Moreover, the σ^54^-dependent promoters realized more rapid and stable production than that of σ^70^-dependent promoters during fed-batch fermentation, producing up to 4.78 g L ^− 1^ of total biofuels.

**Conclusions:**

These results suggested that the nitrogen-responsive transcriptional machinery offers the potential to decouple production from growth, highlighting this system as a novel candidate to realize growth phase-independent and stress-resistant biofuel production.

## Background

**Fig. 1:**
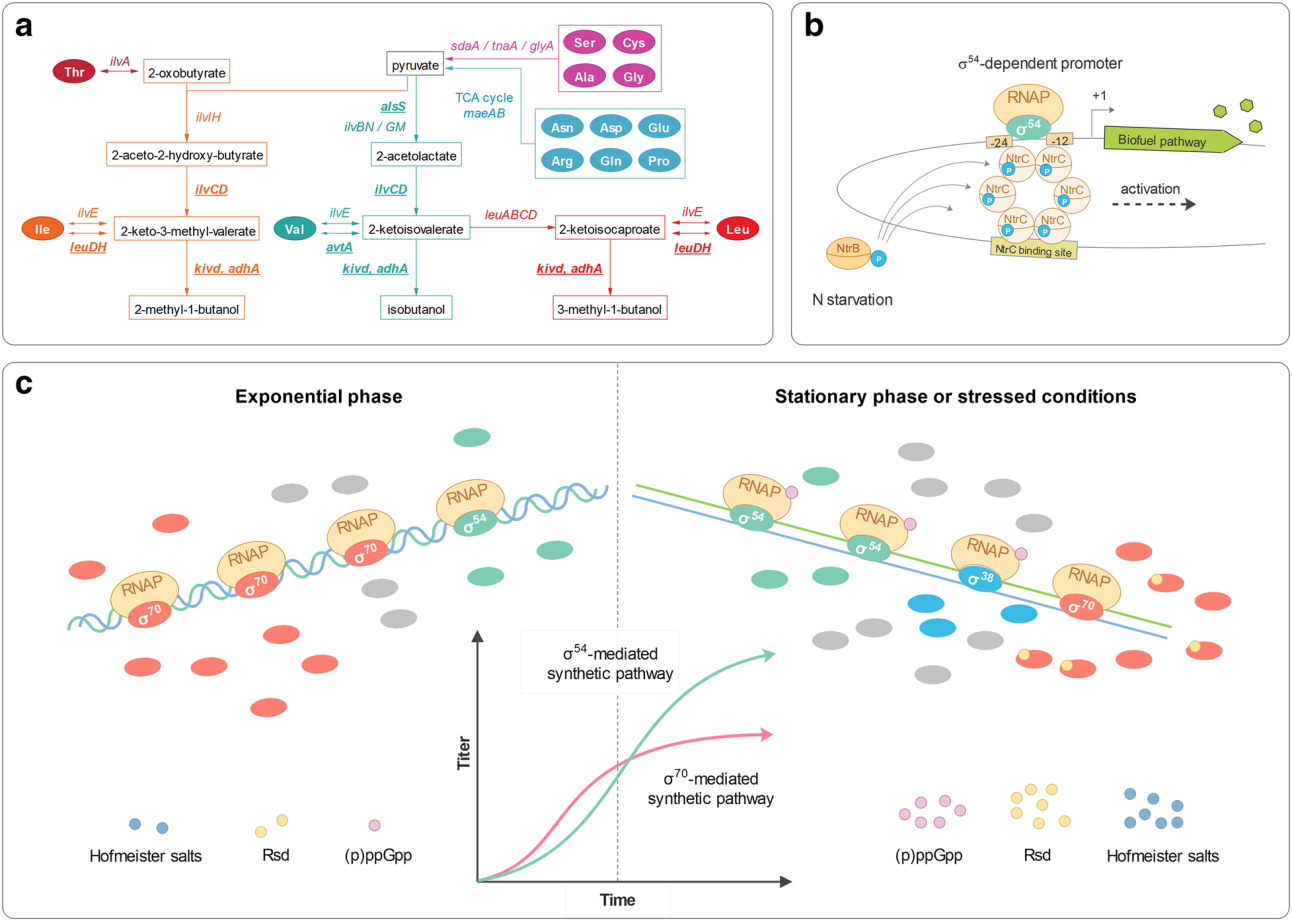
Scheme of the protein-to-fuel conversion driven by the nitrogen-responsive transcriptional machinery. **a** The protein-to-fuel biosynthetic pathways. A total of seven genes are overexpressed for the biosynthesis of higher alcohols from protein biomass. Acetolactate synthase, ketol–acid reductoisomerase, and dihydroxy acid dehydratase are encoded by *alsS* (UniProt: Q04789), *ilvC* (UniProt: P05793), and *ilvD* (UniProt: P05791), respectively, together, these enzymes convert pyruvate to 2-ketoisovalerate (KIV) and 2-keto-3-methyl-valerate (KMV), which are the direct precursors of valine and isoleucine, respectively. A substantial proportion of the produced KIV, KMV, and the leucine precursor 2-ketoisocaproate (KIC) are forced to undergo decarboxylation catalyzed by the 2-ketoisovalerate decarboxylase (encoded by *kivd* (UniProt: Q684J7)) and are then reduced to the corresponding higher alcohols by the alcohol dehydrogenase (encoded by *yqhD* (UniProt: Q46856)), producing isobutanol, 2-methyl-1-butanol, and 3-methyl-1-butanol, respectively. The *leuDH* (UniProt: Q60030), which encodes the leucine dehydrogenase and the *avtA* (UniProt: P09053), encoding the valine–pyruvate aminotransferase, are also overexpressed to drive the amino acid flux into fuel production. With the continuous consumption of the keto acids by the decarboxylase, the reversible reactions catalyzed by LeuDH and AvtA would proceed toward the release of carbon skeletons from amino acids. Other amino acids could be directly transformed into pyruvate through transamination and deamination, or indirectly through the tricarboxylic acid (TCA) cycle, and finally being channeled into biofuel synthesis. **b** Transcription of the σ^54^-dependent promoters mediated by the nitrogen regulatory proteins. Under nitrogen starvation, the phosphorylated NtrB (NtrB-P) transfers its phosphoryl group to NtrC, which interacts with Eσ^54^ and activates the transcription. **c** Assumed performance of the σ^54^-mediated biofuel production throughout the whole growth phase and under stress conditions. *Rsd* regulator of σ^D^, *(p)ppGpp *guanosine pentaphosphate or tetraphosphate

Protein-based biorefining for the production of biofuels and ammonia fertilizer constitutes a promising technology to simultaneously reclaim the carbon and nitrogen from waste proteins [1–5]. To realize protein-to-fuel conversions, the amino acids must be forced to degrade through artificial transamination and deamination cycles in the host organism in order to release the carbon skeletons for biofuel synthesis (Fig. 1a) [3]. However, although currently utilized conversion pathways have been precisely designed, the resultant biofuel productivity remains far below the theoretical level [3, 6]. A major hurdle preventing efficient conversion of proteins into value-added chemicals is the growth-dependency of the production process [4]. As high productivity can only persist in periods of cell growth, but rapidly ceases once the cells enter the stationary phase or encounter stresses, a considerable proportion of the protein source is reallocated to cell growth and maintenance rather than biofuel production. Therefore, to enhance production, we hypothesized that the protein-to-fuel flux could be engineered to resist shifts in the growth phase and the stresses accompanying the fermentation process.

The protein-to-fuel flux is sustained by robust expression of the biofuel synthetic pathway. In general, the engineered biosynthetic pathways in bacteria for the production of value-added chemicals are mostly governed by σ^70^-dependent promoters [7], the transcription of which is determined by the number of the RNA polymerase (RNAP) carrying the σ^70^ subunit (Eσ^70^). Owing to its dominant abundance, this subunit can easily outcompete alternative σ factors for the finite core RNAP [8]. However, the relative advantage of σ^70^ over other σ factors in recruiting the core enzyme is highly compromised once the cells enter the stationary phase or encounter stresses. This derives in part from the sharp increase in the number of alternative σ factors (e.g., σ^38^ and σ^24^) in response to both intra- and extracellular disturbances. In addition, regulatory molecules such as Hofmeister salts, regulator of σ^D^, and guanosine pentaphosphate or tetraphosphate [9, 10] simultaneously accumulate, whereas chromosomal DNA supercoiling decreases [11]. Together, these physiological shifts suppress the association between the core RNAP and σ^70^, facilitating core RNAP interaction with alternative σ factors at the expense of Eσ^70^ [9]. As a result, the protein conversion pathway ceases to function once the fermentation persists to the late stage. To overcome the innate drawbacks of σ^70^-mediated transcription, we posited that metabolic engineering could transform the intrinsic transcriptional regulation process into a driving force for the robust biorefining of waste protein.

In particular, the nitrogen-responsive transcriptional machinery offers a possible solution to achieve growth phase-independent and stress-resistant protein-to-fuel conversion. This machinery consists of the RNAP-σ^54^ holoenzyme (Eσ^54^), the nitrogen regulatory proteins, and the corresponding σ^54^-dependent promoters. For *Escherichia coli*, σ^54^ constitutes one of the most abundant σ factors following σ^70^, and its intracellular concentration tends to remain stable throughout the whole growing stages [12]. In addition, σ^54^ appears to exhibit the highest affinity to the core RNAP among all the alternative σ factors [13] and as the regulatory molecules accumulate, the association between σ^54^ and the core RNAP is further strengthened upon entering into the stationary phase or encountering stresses. In combination, these determinants would be expected to facilitate the formation and maintenance of sufficient Eσ^54^ to support biofuel production under a wide range of physiological conditions.

Moreover, the transcription of σ^54^-dependent promoters is mostly related to the nitrogen status. A limited supply of ammonia can activate σ^54^-mediated transcription, whereas it is inhibited by an increase in the ammonia concentration. This nitrogen-specific response of the σ^54^-dependent promoter is achieved through a cascade of phosphorylation of the nitrogen regulatory proteins such as nitrogen regulatory protein C (NtrC). Under nitrogen starvation conditions, NtrC receives the phosphoryl group from phosphorylated nitrogen regulatory protein B (NtrB) and hydrolyzes ATP, which enables Eσ^54^ to denature the double-stranded DNA and initiate transcription (Fig. 1b). Notably, compared with ammonia, the amino acids support slower growth and are considered to be poor nitrogen sources [14]. The use of amino acids from protein hydrolysate as the sole nitrogen supply can thus cause nitrogen starvation and induce σ^54^-mediated transcription [14, 15]. Therefore, the nitrogen-responsive transcriptional machinery holds promise to resist transcriptional switchover during the stationary phase and under stressed conditions, and should remain active throughout the whole process of protein conversion (Fig. 1c).

Accordingly, in this study we aimed to regulate the expression of the protein conversion pathway to realize robust production of biofuels from protein biomass. Toward this end, the ammonia assimilation pathway in *E. coli* was first engineered to sustain the activities of σ^54^-dependent promoters. To exploit σ^54^-mediated transcription, a series of NtrC-dependent promoters were characterized using a fluorescence reporting system under nitrogen starvation conditions and the promoters exhibiting high activities were identified. Effects of the candidate promoters in maintaining robust protein-to-fuel conversion were evaluated under both optimal and stressed conditions with various protein sources. We postulated that the nitrogen-responsive transcriptional machinery could realize stable pathway expression throughout exponential growth to the stressed stationary phase, rendering the σ^54^-mediated transcriptional machinery a novel candidate to drive robust chemical production from waste proteins.

## Results

### Influence of ammonia assimilation on sustaining σ^54^-mediated transcription

**Fig. 2:**
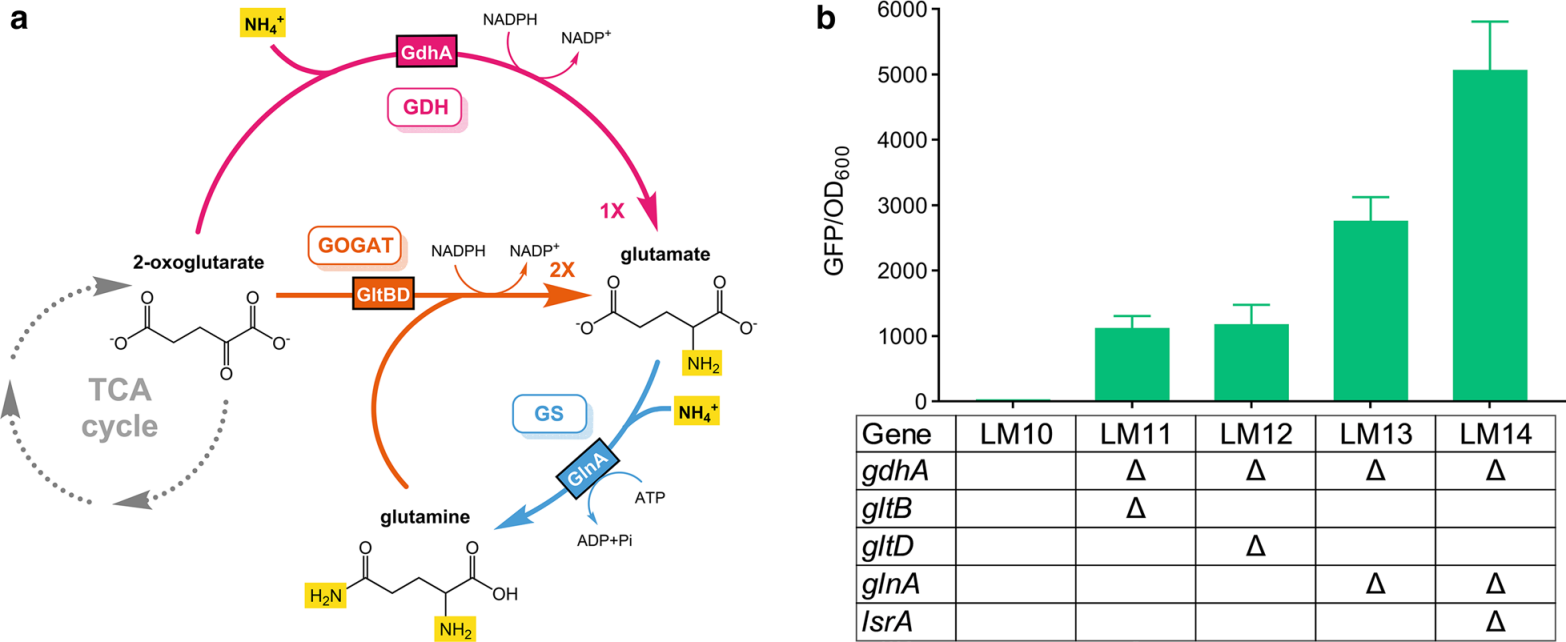
Effects of ammonia assimilation on the activity of the σ^54^-dependent promoter. **a** The ammonia assimilation pathway for *E. coli*. *GDH* glutamate dehydrogenase, *GS* glutamine synthetase, *GOGAT* glutamate synthase. **b** Activities of the σ^54^-dependent promoter *glnAp2* as evaluated by the fluorescence intensities for strains with disrupted ammonia assimilation pathways. Values and error bars represent the mean and the s.d. (n = 3)

In general, to maintain active σ^54^-mediated transcription, the *E. coli* cells must be maintained under nitrogen starvation conditions. However, the deamination of amino acids will release NH_3_, which would be reused by the cells as a preferred nitrogen source; as NH_3_ accumulates, the σ^54^-dependent promoters would cease to function. Therefore, disrupting the intrinsic ammonia assimilation pathway was flagged as the key to achieving continuous transcription from σ^54^-dependent promoters. Genes involved in the glutamate dehydrogenase (GDH) and glutamine synthetase–glutamate synthase (GS–GOGAT) pathways [16] were selectively deactivated (Fig. 2a) and the activity of σ^54^-mediated transcription was measured using a green fluorescence protein (GFP)-based reporting system driven by the typical σ^54^-dependent promoter *glnAp2* in an amino acid-rich environment. As expected, suppressing ammonia assimilation led to sharp increase in the fluorescence intensity (GFP/OD_600_) from nearly 0 (strain LM10) to 2800 (LM13) (Table 1 and Fig. 2b). Disrupting the GDH and GOGAT pathways by knocking out *gdhA* together with *gltB* or *gltD* led to similar fluorescence intensity levels of around 1100, as shown for LM11 and LM12, respectively. In comparison, disruption of the GS instead of the GOGAT pathway in a GDH-deficient background achieved a 2.4-fold increase in the fluorescence intensity (LM13).

**Table 1:** Plasmids and strains used in this study

Plasmids	Description	Origin	Resistance	References
pSA69	*P*_L_lacO_1_: *alsS-ilvC-ilvD*	p15A	Kanamycin	[17]
pYX97	*P*_L_lacO_1_: *leuDH-kivD-yqhD; lacI*	colE1	Ampicillin	[3]
pLM1	*P*_L_lacO_1_: *alsS-ilvC-ilvD-avtA*	p15A	Kanamycin	This study
pLM2	*rrnBp1*: *alsS-ilvC-ilvD-avtA*	p15A	Kanamycin	This study
pLM3	*rrnBp1*: *leuDH-kivD-yqhD; lacI*	colE1	Ampicillin	This study
pLM4	J23100: *alsS-ilvC-ilvD-avtA*	p15A	Kanamycin	This study
pLM5	J23100: *leuDH-kivD-yqhD; lacI*	colE1	Ampicillin	This study
pLM6	*glnAp2*: *alsS-ilvC-ilvD-avtA*	p15A	Kanamycin	This study
pLM7	*glnAp2*: *leuDH-kivD-yqhD; lacI*	colE1	Ampicillin	This study
pLM8	*argTp*: *alsS-ilvC-ilvD-avtA*	p15A	Kanamycin	This study
pLM9	*argTp*: *leuDH-kivD-yqhD; lacI*	colE1	Ampicillin	This study
pLMg1	*argTp*: *gfp*	p15A	Kanamycin	This study
pLMg2	*astCp*: *gfp*	p15A	Kanamycin	This study
pLMg3	*ddpXp*: *gfp*	p15A	Kanamycin	This study
pLMg4	*glnHp2*: *gfp*	p15A	Kanamycin	This study
pLMg5	*glnKp*: *gfp*	p15A	Kanamycin	This study
pLMg6	*nacp*: *gfp*	p15A	Kanamycin	This study
pLMg7	*pabB*: *gfp*	p15A	Kanamycin	This study
pLMg8	*patAp*: *gfp*	p15A	Kanamycin	This study
pLMg9	*puuPp*: *gfp*	p15A	Kanamycin	This study
pLMg10	*rutAp*: *gfp*	p15A	Kanamycin	This study
pLMg11	*yhdWp*: *gfp*	p15A	Kanamycin	This study

Strain	Description	References
XL10-Gold	Tet^r^Δ(*mcrA*)*183* Δ(*mcrCB-hsdSMR-mrr*)*173 endA1 supE44 thi-1 recA1 gyrA96 relA1 lac* Hte [F´ *proAB lacI*^q^*Z*Δ*M15* Tn*10* (Tet^r^) Amy Cam^r^]	Agilent technologies
JCL16	BW25113/F’ [*traD*36, *proAB*^+^, *lacI*^q^*Z*Δ*M15*	[18]
LM10	A JCL16 mutant with enhanced ability to utilize amino acids	[3]
LM11	LM10 with Δ*gdhA*, Δ*gltB*	This study
LM12	LM10 with Δ*gdhA*, Δ*gltD*	This study
LM13	LM10 with Δ*glnA*, Δ*gdhA*	This study
LM14	LM10 with Δ*glnA*, Δ*gdhA*, Δ*lsrA*	This study
LM15	LM14 with plasmids pYX97 and pLM1	This study
LM16	LM14 with plasmids pLM2 and pLM3	This study
LM17	LM14 with plasmids pLM4 and pLM5	This study
LM18	LM14 with plasmids pLM6 and pLM7	This study
LM19	LM14 with plasmids pLM8 and pLM9	This study

To further enhance the transcriptional activity, the quorum sensing (QS) pathway, which has the potential to increase strain robustness under stressed conditions [3], was blocked by deleting *lsrA*, which encodes the transporter for autoinducer-2. This GDH-, GS-, and QS-deficient strain (LM14) exhibited 1.8-fold increase in *glnAp2*-mediated transcription compared with that of LM13 (Fig. 2b). When amino acids were supplied as the sole nitrogen source, the maximum OD_600_ of LM14 was half that of LM10. The growth rate of LM14 at the exponential phase was 15 to 47% that of LM10 (Additional File 1: Figure S1). As a result, more resources in LM14 were allocated to biofuel production than that of LM10 (Additional File 1: Figure S2).

### Mining of σ^54^-dependent promoters

**Fig. 3:**
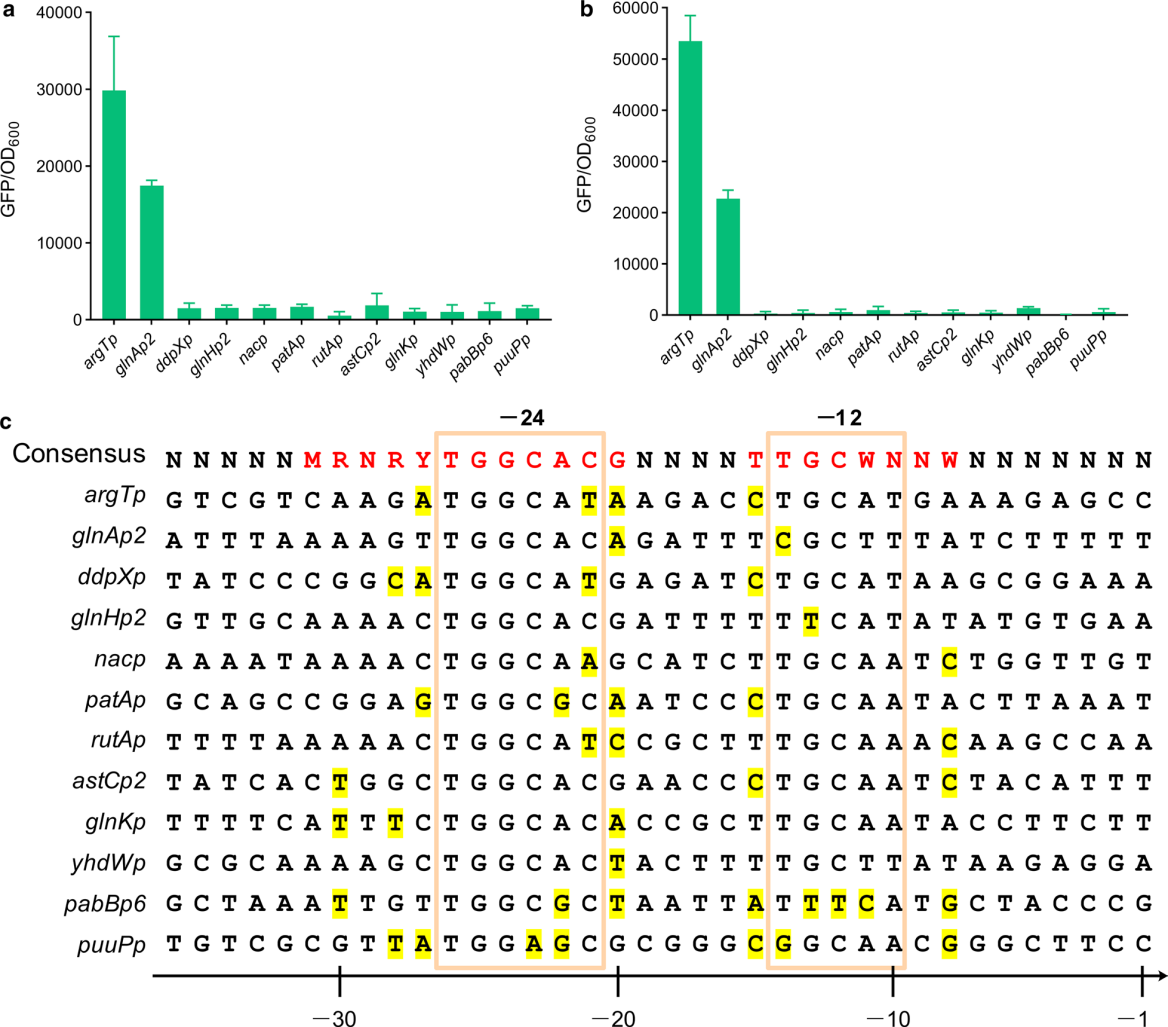
Fluorescence intensities for cells expressing GFP from different σ^54^-dependent promoters. **a**, **b** Fluorescence intensities for cells in the exponential phase and the stationary phase, respectively. **c** Sequence alignment of the selected promoters. The − 12 and − 24 elements are boxed. Nucleotides in red represent the consensus sequence of the σ^54^-dependent promoters. Nucleotides that differ from the consensus sequence are shaded. Values and error bars represent the mean and the s.d. (n = 3)

To identify σ^54^-dependent promoters that are able to maintain high transcriptional activity under nitrogen-limited conditions, a total of 12 σ^54^-dependent promoters (Additional file 1: Table S1), of which 10 were regulated by the nitrogen regulator NtrC, were each inserted upstream of *gfp* and characterized based on fluorescence intensity. When yeast extract was used as the sole nitrogen source, strains harboring different *gfp* expression cassettes exhibited fluorescence intensities ranging from 538 to 29,836 in the exponential phase. The *gfp* expression driven by *argTp* showed the highest intensity, which was 1.7-fold that of *glnAp2* and 17–56 times higher than that of the remaining candidates (Fig. 3a). The transcriptional activities for *argTp* and *glnAp2* were strengthened when cells entered the stationary phase (Fig. 3b). Compared with the exponential phase, 1.8- and 1.3-fold increases in the fluorescence intensities for *argTp* and *glnAp2* were observed, respectively. The superior activity of *argTp* was confirmed by repeated measurement (Additional file 1: Figure S3), for which the fluorescence intensity generated from the *argTp*-*gfp* construct was 2.7–7.5 times higher than that of the remaining candidates. However, the fluorescence intensity produced by the *glnAp2*-*gfp* construct was comparable to that of the remaining promoters upon assay repetition. Nevertheless, to cover as many potential candidates as possible, both *argTp* and *glnAp2* were tested for their performance with regard to driving protein-to-fuel conversion.

Sequence alignment showed that seven promoters including *argTp* and *glnAp2* contained − 12/− 24 elements identical to the previously defined − 12/− 24 consensus sequences [19] (Fig. 3c) and three promoters including *astCp2*, *glnKp*, and *yhdWp* exhibited perfect matches with the consensus, whereas *puuPp* and *pabBp6* displayed 1–3 mismatches in both their − 12 and − 24 elements. Mismatches in the extended − 12/− 24 regions (− 15 to − 8 and − 31 to − 20) in comparison to the consensus sequences are also presented in Fig. 3c.

### Biofuel production driven by the σ^54^-dependent promoters

**Fig. 4:**
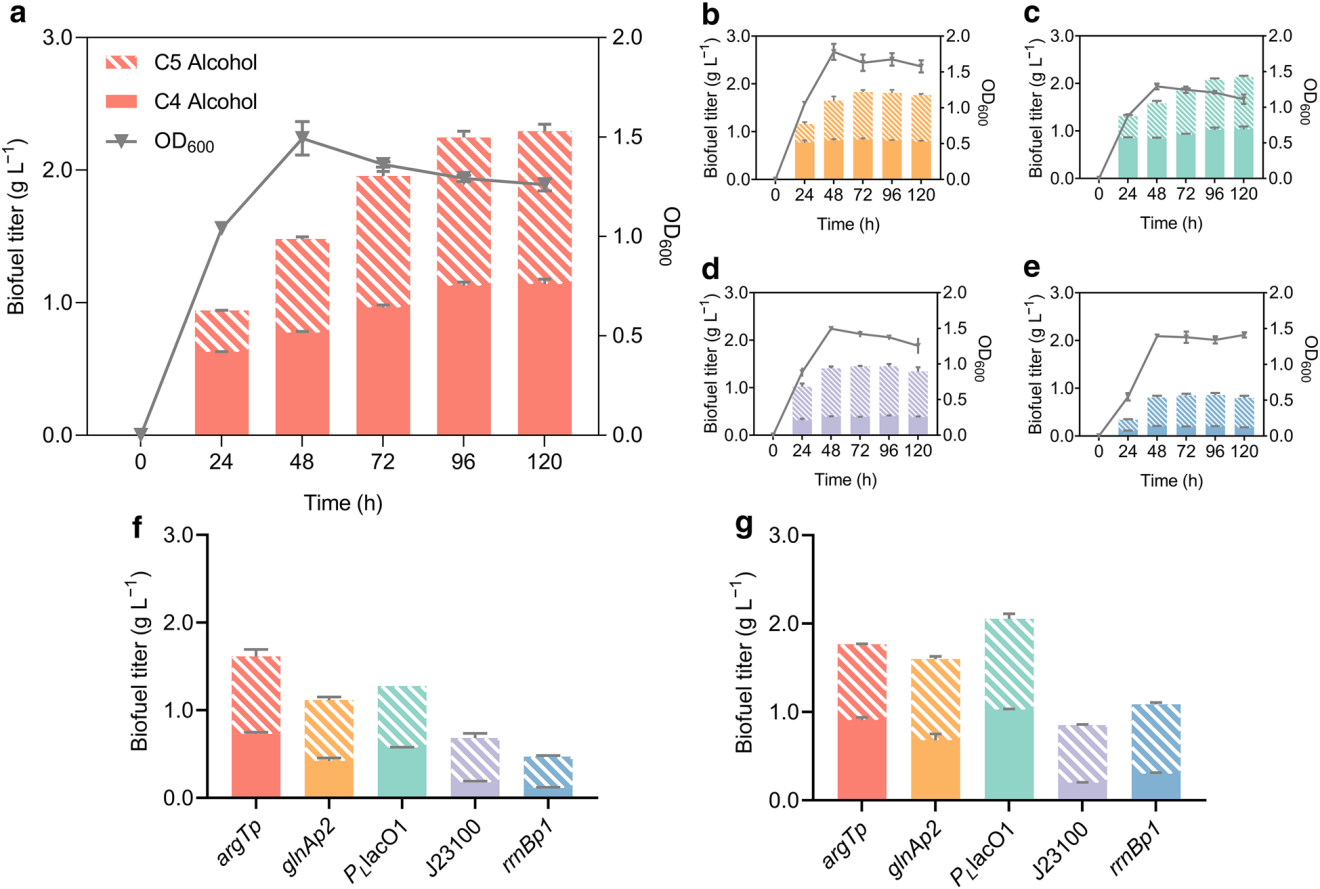
Biofuel production driven by either the selected σ^54^-dependent promoters or the commonly used σ^70^-dependent promoters. **a** Biofuel production for strain LM19 with promoter *argTp* under the optimal condition. **b** Biofuel production for strain LM18 with promoter *glnAp2*. **c** Biofuel production for strain LM15 with promoter *P*_L_lacO_1_. **d** Biofuel production for strain LM17 with promoter J23100. **e** Biofuel production for strain LM16 with promoter *rrnBp1*. **f** Biofuel production under conditions of osmotic stress (400 mM NaCl). **g** Biofuel production under conditions of acid stress (pH 5.0). Values and error bars represent the mean and the s.d. (n = 3)

The effect of σ^54^-dependent promoters in driving protein-to-fuel conversion was investigated in comparison with that of three σ^70^-dependent promoters including *rrnBp1*, J23100, and *P*_L_lacO_1_. *rrnBp1* constitutes a typical σ^70^-dependent promoter that drives the bulk transcription of ribosomal DNA [20]. J23100 is a strong synthetic promoter (iGEM Part: BBa J23100) and *P*_L_lacO_1_ is a commonly used promoter for biofuel production [3, 17]. Isobutanol (C4) and methylbutanols (2-methyl-1-butanol and 3-methyl-1-butanol, C5) were produced through the synthetic pathway. For all promoters, the total biofuel titers increased sharply in the first 48 h when cells were in the exponential phase (Fig. 4a–e). Upon entering into the stationary phase, the biofuel production from *rrnBp1* and J23100 ceased rapidly (Fig. 4d, e). In comparison, the biofuel titer for strain LM19 with promoter *argTp* continued to increase at a steady rate during the stationary phase with a total of 0.84 g L^− 1^ biofuel being produced from 48 to 120 h (Fig. 4a), which was 1.7-fold of the corresponding net biofuel production for strain LM15 with promoter *P*_L_lacO_1_ (Fig. 4c). *argTp*, *glnAp2*, and *P*_L_lacO_1_ promoters produced similar amounts of the C4 and C5 alcohols in the final products, whereas the C5 alcohol dominated the produced biofuels for J23100 and *rrnBp1*. The σ^70^-dependent promoters achieved final titers of 0.17 to 1.03 g L^− 1^ for C4 alcohol and 0.63 to 1.13 g L^− 1^ for C5 alcohol, whereas higher titers of 0.79 to 1.14 and 0.97 to 1.36 g L^− 1^ were achieved for C4 and C5 alcohols, respectively, by the σ^54^-dependent promoters. In total, the pathway driven by *argTp* produced 2.50 g L^− 1^ biofuels, which was 16% higher than that of *P*_L_lacO_1_. *glnAp2* also showed advantage over the σ^70^-dependent promoters by achieving a final titer 1.3- to 2.2-fold higher than that of J23100 and *rrnBp1*.

The performance of σ^54^-mediated biosynthesis was also evaluated under stressed conditions. Compared with optimal conditions, osmotic stress induced by 400 mM NaCl suppressed biofuel production of the σ^70^-dependent promoters by 41% to 49% (Fig. 4f). In comparison, the σ^54^-dependent promoter sustained its advantage in biofuel production. *argTp* achieved the highest biofuel production of 1.61 g L^− 1^, which was 26% higher than that of *P*_L_lacO_1_. A titer equivalent to 1.6- and 2.4-fold that of J23100 and *rrnBp1*, respectively, was also achieved by *glnAp2*. Moreover, stress related to sharply decreased pH may also arise consequent to the pretreatment of protein biomass. Compared with the σ^70^-dependent promoters, *argTp* retained its advantage in driving pathway overexpression under an initially acidified (pH 5.0) condition, leading to one of the highest biofuel titers of 1.77 g L^− 1^ (Fig. 4g). The biofuel production driven by *glnAp2* also exceeded that of J23100 and *rrnBp1* by 2.1- and 1.5-fold, respectively.

The biofuel synthetic pathway driven by *argTp* produced more pathway enzymes than that driven by the σ^70^-dependent promoters in both the optimal and the stressed conditions. When fermentation proceeded to the stationary phase under optimal conditions, the activity of the acetolactate synthase (AlsS) enzyme expressed via the *argTp* promoter was 42% higher than that from *P*_L_lacO_1_ (Additional file 1: Figure S4a). Under osmotic (Additional file 1: Figure S4b) or acid stresses (Additional file 1: Figure S4c), the activity of AlsS expressed via *argTp* remained 34% and 93% higher than that from *P*_L_lacO_1_, respectively, and was 1.5- to 3.5-fold that of the remaining σ^70^-dependent promoters.

### Batch fermentation driven by the σ^54^-dependent promoters

To mimic the industrial fermentation process, a fed-batch fermentation was carried out in the presence of oleyl alcohol to extract the produced biofuel from the aqueous phase (Additional file 1: Figure S5). The σ^54^-dependent promoters realized a rapid and stable production of biofuels, with a longer producing period and a higher final titer than those of σ^70^-dependent promoters. In the exponential phase, the σ^54^-dependent promoters synthesized the biofuels at a rate of 47.81 to 49.81 mg L^− 1^ h^− 1^ with the titer reaching 2.29 to 2.39 g L^− 1^. After entering the stationary phase, these promoters continued to produce at a rate of 10.47 to 14.22 mg L^− 1^ h^− 1^ for 168 h, with the final titer reaching 4.05 to 4.78 g L^− 1^. In contrast, the σ^70^-dependent promoters produced biofuels at a rate of 35.69 to 42.26 mg L^− 1^ h^− 1^ during the exponential phase. However, the production rate dropped sharply by 66 to 77% upon entering the stationary phase and then decreased to zero after 72 h. The final titer achieved by σ^70^-dependent promoters was 75 to 88% that of the σ^54^-dependent promoters.

### Biofuel production from waste protein biomass

**Fig. 5:**
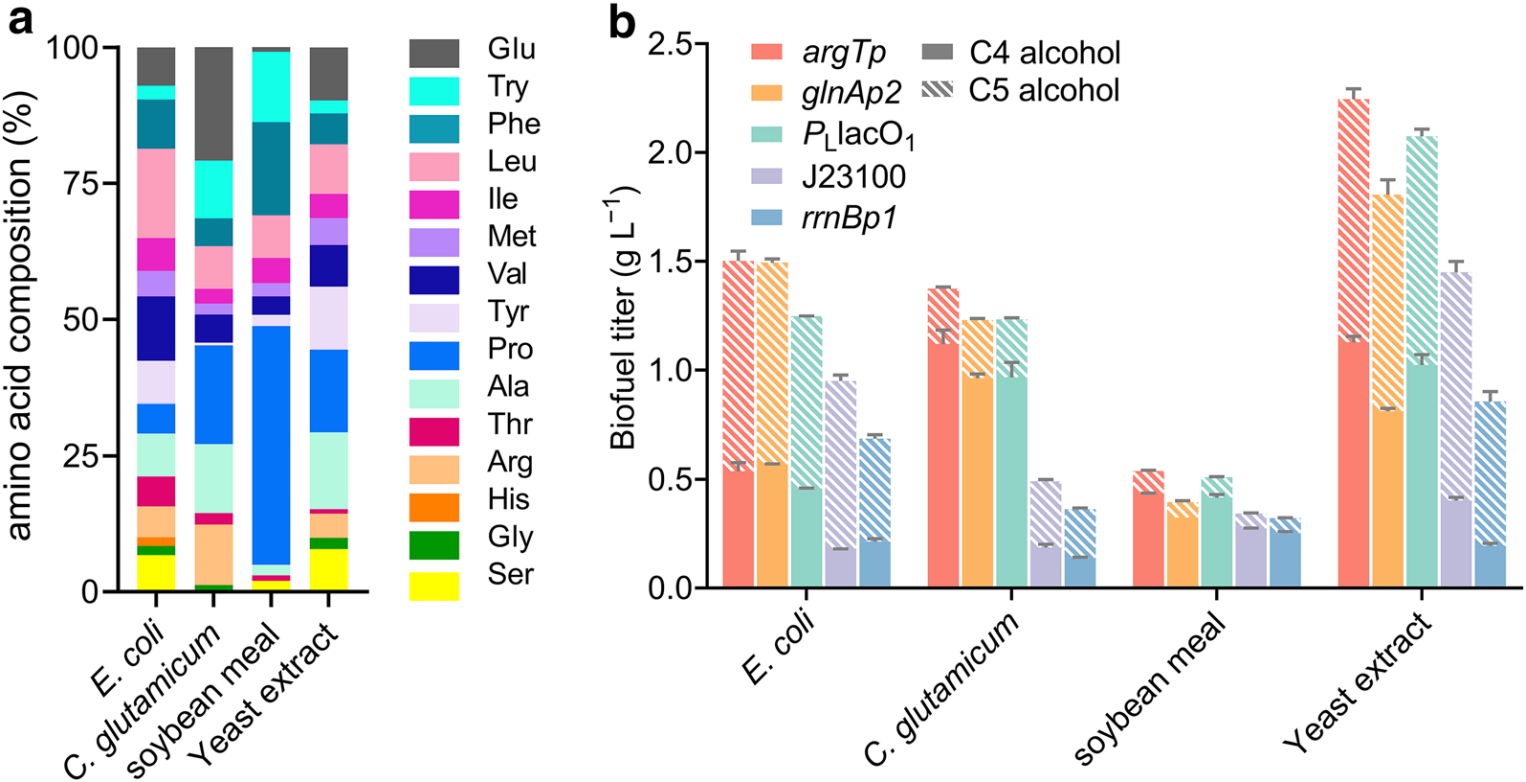
Biofuel production from protein biomass. **a** Amino acid compositions of different protein sources. **b** Biofuel production from protein biomass driven by different promoters. Values and error bars represent the mean and the s.d. (*n* = 3)

To examine the performances of the σ^54^-dependent promoters in driving waste protein conversion, microbial proteins from *E. coli* and *Corynebacterium glutamicum* cells, and plant proteins from soybean meal were used as feedstock for biofuel production (Fig. 5a). A total of 0.32 to 1.55 g L^− 1^ higher alcohols were produced using these protein biomasses. When *E. coli* biomass was supplied, 62 to 81% of the produced biofuel comprised C5 alcohol, whereas for *C. glutamicum* biomass, the main portion of the biofuel changed to C4 alcohol. Plant proteins produced lower amounts of biofuels ranging from 0.32 to 0.54 g L^− 1^, of which the majority constituted C4 alcohol, which accounted for 78 to 82% of the total biofuel (Fig. 5b). The highest biofuel production was achieved when yeast protein was used as the feedstock, with C5 alcohol representing 50 to 77% of the titer. In general, pathways driven by the σ^54^-dependent promoters produced more biofuel than those driven by the σ^70^-dependent promoters. *argTp* demonstrated advantage over the other promoters for fermentation with all feedstocks. On average, the biofuel production driven by *argTp* was 1.41 g L^− 1^, which was up to 2.53-fold that the production driven by σ^70^-dependent promoters. Pathways driven by the σ^54^-dependent promoters also produced higher proportions of C4 alcohol in the total biofuel than those from the σ^70^-dependent promoters.

## Discussion

In this study, we demonstrate that the nitrogen-responsive transcriptional machinery in *E. coli* is able to drive robust biofuel production from protein biomass. Compared with the commonly used σ^70^-dependent promoters, σ^54^-mediated biofuel production could retain high productivity in the stationary phase and achieve higher biofuel titers under both optimal and stressed conditions. With simple modifications of the ammonium assimilation pathways and the use of suitable promoters, the σ^54^-mediated scheme therefore offers a promising alternative to the conventional σ^70^-mediated approach for the biorefining of waste proteins.

The nitrogen-responsive transcriptional machinery provides multiple advantages over the conventional σ^70^-mediated scheme in driving protein conversion [3, 17]. The first is extension of the period supporting biosynthetic activity, as the pathway driven by *argTp* retained a relatively high biofuel productivity from early to late stationary phases, whereas that driven by σ^70^-dependent promoters basically ceased to function upon completion of cell growth. In particular, although the σ^70^-dependent promoters could only retain activity for 48–72 h after entering the stationary phase, the σ^54^-dependent promoters could function for more than 168 h (Additional file 1: Figure S5). Notably, maintaining production in the stationary phase is of marked significance for the industrial production of chemicals. For example, the cell factories face trade-offs between growth and production. When cells enter the stationary phase, the resources dedicated for growth are minimized and could be rechanneled for production; thus, this represents an optimal stage to boost overall yield. For this purpose, the concept of decoupling growth and production has been proposed and considered as a grand challenge for metabolic engineering [21, 22]. In the present study, *glnAp2* and *argTp* reached the highest yield when using microbial protein biomass as the feedstock, achieving 17% of the theoretical level (Additional file 1: Figure S6), and was up to 2.93-fold that of the σ^70^-dependent promoters. Therefore, improving productivity in the stationary phase appears to be beneficial for industrial processes. Moreover, most industrial production consists of continuous batch fermentation, in which the majority of production occurs during the stationary phase [23]. In batch fermentation, the strain carrying the σ^70^-dependent promoter could only maintain productivity up to 11.13 mg L^− 1^ h^− 1^ after entering the stationary phase, whereas the σ^54^-dependent promoter retained a productivity 28% higher throughout the whole stationary phase (Additional file 1: Figure S5).

The nitrogen-responsive transcriptional machinery also avoids the utilization of inducers, which are essential for the biosynthetic pathways governed by inducible σ^70^-dependent promoters such as the isopropyl β-d-1-thiogalactopyranoside (IPTG)-induced *P*_L_lacO_1_. The exclusion of chemical inducers reduces the fermentation cost and is more feasible for industrial scale-up of the protein-based biorefinery. Additionally, as the NtrC-mediated promoters mainly respond to nitrogen starvation, their transcription can be strengthened through depletion of the nitrogen source, as fermentation persists to the late stages. This intrinsic driving force can compensate for the reduced pathway expression resulting from the stationary phase and accompanying stresses, endowing *argTp* and *glnAp2* equivalent or even superior performance than that of the strong inducible promoter *P*_L_lacO_1_.

The resistance to general stresses such as high osmolarity and low pH constitutes another advantage, as these can significantly inhibit biofuel production driven by σ^70^-dependent promoters [24, 25]. It should be noted that for protein-based biosynthesis, the pH of the medium is less likely to decrease during the fermentation due to the continuous release of NH_3_ from amino acid deamination. However, acid stress can still stem from pretreatment processes, such as purification and hydrolysis of the protein biomass [1]. Therefore, the stress resistance endowed by σ^54^-mediated biosynthesis may further promote robust biofuel production from waste proteins. The biofuel titers achieved in this study did not exceed those previously reported [3]. This may be largely attributed to differences in the yeast extract used for feeding, for which the amino acid concentration was only half that of the yeast extract used in other studies [3, 6].

Maintaining nitrogen starvation constitutes an important prerequisite to induce stable transcription of the nitrogen-responsive promoters. When using amino acids as the feedstock, the knockout of both the GDH and GS pathways could create a nitrogen-limited intracellular status under an ammonia-rich environment, thereby achieving high activities of the NtrC-mediated promoters. This specific phenotype could be inferred from the regulatory cascade in nitrogen assimilation (Additional file 1: Figure S7). For many bacteria, the nitrogen status is sensed by the bifunctional uridylyltransferase/uridylyl-removing enzyme (GlnD) and PII signal transduction systems. In *E. coli*, GlnD responds to intracellular glutamine and PII is regulated by 2-oxoglutarate [26]. The accumulation of glutamine leads to the dephosphorylation of phosphorylated NtrC (NtrC-P) and suppresses NtrC-mediated transcription. In contrast, excessive 2-oxoglutarate promotes the release of free NtrB and subsequently increases the level of NtrC-P and activates the targeted transcription. In general, a high 2-oxoglutarate to glutamine ratio indicates nitrogen starvation [16, 27], which signals the cell to activate nitrogen assimilation pathways controlled by the NtrC. In this case, the conversion of 2-oxoglutarate to glutamate is prevented owing to the deficiency of GDH, and the production of glutamine from glutamate is also blocked after knocking out the GS. This results in the accumulation of 2-oxoglutarate and consumption of glutamine, leading to an increased ratio between these two effectors and thus deceiving the cells into maintaining high transcriptional activities of the NtrC-mediated promoters. Upon deletion of the ammonia assimilation pathway, the engineered strain can no longer reuptake the newly produced NH_3_, which limits its growth. However, when equipped with a σ^54^-dependent promoter, the LM19 strain could reallocate more resources dedicated to growth instead to production, achieving 3.16-fold higher biofuel production than that of the LM10 strain equipped with biofuel synthetic pathways (Additional file 1: Figure S2).

Disruption of QS further enhanced protein expression driven by the σ^54^-dependent promoters. When cells enter the stationary phase or encounter stresses, the QS signaling molecule autoinducer-2 (AI-2) accumulates [28], causing DNA damage and metabolic shifts [29, 30]. Therefore, blocking AI-2 uptake by deleting *lsrA*, which encodes the ATP-binding component of the AI-2 transporter, could prevent the cell population from collectively switching their metabolic state from biosynthesis to maintenance, thereby leading to robust chemical production throughout the whole growth phase.

Analysis of the σ^70^-dependent promoters suggested that inclusion of the σ binding sites most resembling the consensus sequence would facilitate promoter opening and the formation of an open complex, thereby increasing the transcriptional strength of the promoters [31]. However, this appeared not to be the case for the 12 σ^54^-dependent promoters evaluated in the present study, as promoters (e.g., *astCp2*, *glnKp*, and *yhdWp*) exhibiting perfect matches to the − 12 and − 24 consensus sequences basically showed the lowest transcriptional activities throughout the exponential to the stationary phases (Fig. 3). In comparison, although containing mismatches in the − 12 or − 24 element to the consensus sequence, both the *argTp* and *glnAp2* promoters exhibited dominant transcriptional activities in both the exponential and stationary phases. Therefore, the resemblance of the core promoter elements to their consensus sequences might not constitute a reliable criterion for identifying strong σ^54^-dependent promoters. The lack of a close relationship between the transcriptional strength and the degree of sequence conservation for the σ^54^-dependent promoters may be attributed to the involvement of activators, which play additional roles in initiating σ^54^-dependent transcription. Taking this into consideration, sequences in the upstream region of the core promoter elements may be crucial for activator binding, its orientation with regard to Eσ^54^ [32], and the DNA looping that leads to the formation of the activator–Eσ^54^ complex [33]. In the present case, the NtrC binding sites on *argTp* and *glnAp2* might provide the proper binding strength and favored relative positioning of NtrC-P toward Eσ^54^. In addition, the sequences in between the NtrC binding sites and the core promoter region may also produce the right angle of DNA bending to facilitate the NtrC–Eσ^54^ interaction. Therefore, the outstanding performance of *argTp* and *glnAp2* could likely be attributed to the combined effects of the core promoter regions, NtrC binding sites, and intervening bending regions. Moreover, transcription from *argTp* has been shown to increase with procession of the stationary phase [34], which supports the use of *argTp* to drive robust pathway expression.

Our results demonstrated that the composition of produced alcohol is affected by two factors, the amino acid composition of the protein biomass and the dominance of the biofuel synthetic pathway. As the precursors of higher alcohols, branched-chain amino acids (BCAAs) including valine, leucine, and isoleucine can be directly converted into the corresponding alcohols. Thus, the relative abundance of BCAAs in the raw material could directly affect the composition of the produced biofuels. In addition, driven by the biofuel synthetic pathway, the central metabolite pyruvate would be mainly converted to C4 alcohol [17]. Therefore, the more pyruvate is converted from amino acids other than BCAAs, the more C4 alcohol would be produced. However, as pyruvate might be channeled to various pathways, the metabolic flux to biofuels relies on the strength of the biofuel synthetic pathway. A strong and stable promoter would drive the continuous expression of the biofuel synthetic pathway, converting more pyruvate to C4 isobutanol. This may be the reason that the proportion of the produced C4 alcohol in the total biofuel driven by *argTp* and *glnAp2* exceeded that of the σ^70^-dependent promoters. However, it should be noted that both the C4 and C5 alcohols are ideal alternatives to traditional gasoline [17]. Therefore, the composition of the final products has little effect on the quality of the produced biofuels.

By targeting protein bioconversions, this study demonstrated that the nitrogen-responsive transcriptional machinery can be employed for chemical production and has the potential to realize growth phase-independent and stress-resistant overexpression of the biosynthetic pathways. Constructing robust microbial cell factories is essential for achieving higher productivity and represents an important challenge for metabolic engineering [35]. σ^54^-mediated transcriptional regulation offers a convenient solution as it requires only the substitution of a single promoter and small modification of the ammonia assimilation pathway. Subsequent engineering of the host strain, reconstruction of the synthetic pathway, and optimization of the fermentation process might also be performed to further enhance the production. Considering the wide distribution of σ^54^-dependent promoters in prokaryotes [36, 37], the σ^54^-mediated regulation scheme might be further exploited by mining the natural promoter libraries, as for *E. coli* in particular, nearly one hundred σ^54^-dependent promoters have already been experimentally identified or predicted according to the latest collections in RegulonDB [38]. To expand the transcriptional strength and dynamic range of σ^54^-mediated transcription, synthetic σ^54^-dependent promoters might also be generated by engineering the naturally occurring − 12/− 24 elements, spacer region, activator binding sites, or the sequence responsible for DNA looping. These endeavors would facilitate the fine-tuning of the σ^54^-mediated biosynthetic pathways.

The waste proteins as feedstock can be supplied in various forms including microbial, plant, and animal biomass for the σ^54^-mediated biorefineries. In addition to the abundant sources of feedstock, another advantage is that the σ^54^-mediated biosynthetic scheme could theoretically be applied for the production of a myriad of value-added amino acid derivatives (Additional file 1: Figure S8), such as carbocyclic aromatic compounds derived from the shikimate pathway for aromatic amino acid biosynthesis. However, it should be noted that efficient protein-based biorefinement relies upon rewiring the transamination network that channels different amino acids into specific amino acid precursors. Therefore, the design of the transamination network should consider the amino acid compositions of the source materials (Additional file 1: Figure S9) and the corresponding amino acid synthetic pathways for the targeted products. Combined with the σ^54^-dependent promoters, the engineered transamination and the biosynthetic pathway would be expected to promote efficient recycling of both the ammonia and carbon skeletons from waste proteins.

Theoretically, the σ^54^-mediated biosynthetic scheme is not limited to protein conversion. When the nitrogen supply is tightly controlled, σ^54^-mediated transcription can remain active throughout the whole growth stage. Therefore, when materials other than proteins are used as the feedstock, the amino acids may serve as the poor nitrogen source rather than ammonia. As a result, the low availability of the amino acids would trigger the NtrC-mediated promoters and achieve the robust conversion of renewable sources into value-added chemicals.

## Conclusions

Overall, our findings demonstrated that biosynthesis mediated by the nitrogen-responsive transcriptional machinery outcompetes the conventional σ^70^-mediated scheme by enhancing the robustness and productivity of the biosynthetic pathways, rendering it a novel solution to realize growth phase-independent and stress-resistant protein-to-fuel conversion.

## Methods

### Strains and plasmids

A previously reported *E. coli* strain with enhanced amino acid utilization was used for the conversion of proteins into biofuels (Table 1). The derivative strains with gene deletions including *gdhA*, *gltB*, *gltD*, or *lsrA* were created by using P1 transduction or λ phage recombination. Two adjacent promoters were located upstream of *glnA*: the σ^54^-dependent *glnAp2* and the σ^70^-dependent *glnAp1*. The latter was located between the two NtrC binding sites at the 5′ end of *glnAp2*. To eliminate the interference from *glnAp1* on σ^54^-mediated transcription, only the − 1 to − 99 region that encompasses the core *glnAp2* promoter and its first three NtrC binding sites from the 5′ end was cloned from *E. coli* MG1655 genomic DNA. Other σ^54^-dependent promoters were also cloned from the genomic DNA and inserted into the 5′ end of the two gene cassettes comprising the biofuel biosynthetic pathway [3] using Gibson assembly (Additional file 1: Tables S1 and S2). Cloning was carried out using *E. coli* strain XL10-Gold (Agilent Technologies, Santa Clara, CA, USA). The *E. coli* strains were routinely cultured in Luria–Bertani (LB) broth or LB agar supplemented with 50 μg mL^− 1^ kanamycin or 100 μg mL^− 1^ ampicillin.

### Medium and fermentation

Amino acid medium used for biofuel production comprised 40 g L^− 1^ yeast extract (amino acid profile shown in Fig. 5a) with M9 salt containing 6.0 g L^− 1^ Na_2_HPO_4_, 3.0 g L^− 1^ KH_2_PO_4_, 0.5 g L^− 1^ NaCl, 0.12 g L^− 1^ MgSO_4_, 11 mg L^− 1^ CaCl_2_, and 1.0 mg L^− 1^ vitamin B1. Ampicillin (100 μg mL^− 1^) and kanamycin (50 μg mL^− 1^) were added when required. The *E. coli* and *C. glutamicum* grown in LB medium were used as microbial protein sources and soybean meal was used as the plant protein source. The bacterial cells were treated by ultrasonication at 500 W for 40 min, whereas the soybean meal was first autoclaved at 121 °C for 20 min and then disrupted ultrasonically. Released proteins were measured using the Bradford assay and hydrolyzed overnight by protease (120 U mg^− 1^) at a concentration of 4 g kg^− 1^ (dry weight). The produced free amine groups were then quantified using the ninhydrin reaction. For all protein sources, the total amount of peptides and amino acids used for fermentation was adjusted to 12.8 g L^− 1^, which is equivalent to the protein concentration in 40 g L^− 1^ yeast extract (Angel Yeast Co., Ltd., Yichang, Hubei, China). For biofuel fermentation, the overnight seed culture was prepared in 5 mL LB medium at 37 °C in a shaker at 250 rpm. The culture was inoculated at 1% into 20 mL of amino acid medium in a 250 mL screw-cap conical flask. Fermentation was performed in triplicate at 30 °C in a shaker at 250 rpm. The cell OD and the concentrations of higher alcohols were measured at defined time intervals. For continuous fermentation, an equal volume of oleyl alcohol was added to the flask prior to incubation for extraction of the produced isobutanol. Following inoculation, IPTG was added to a final concentration of 0.1 mM. Fermentation was performed in triplicate at 30 °C in a shaker (250 rpm). The aqueous and organic phases were sampled (5 mL each) at defined time intervals. After sampling, the fermentation broth was replenished with fresh medium and oleyl alcohol. All amino acids mentioned in this study were l-amino acids except glycine, which has no chirality.

### Assay of promoter strength

Cells were cultured in 5 mL LB medium at 37 °C in a shaker at 250 rpm. The overnight culture was inoculated at 1% into 200 μL amino acid medium in a 96-well plate, which was sealed with breathable film and incubated at 37 °C. At defined time points, the fluorescence was measured using a Cytation Hybrid Multi-Mode Reader (BioTek, Winooski, VT, USA) with the excitation and detection wavelengths set at 400 and 508 nm, respectively; the OD_600_ was also measured. The ratio of fluorescence to OD_600_ (GFP/OD_600_) was used to represent the promoter strength. The background fluorescence was measured using a strain harboring a promoterless plasmid that carries the *gfp* gene.

### Enzyme assays

The activity of AlsS was measured according to Atsumi [39]. Briefly, 50 μL crude cell extract was mixed with 150 μL MOPS buffer (pH 7.0) containing 100 mM MOPS, 20 mM sodium pyruvate, 0.1 mM thiamine pyrophosphate, and 1 mM MgCl_2_. The enzyme reaction was carried out at 37 °C for 30 min and terminated by adding 20 μL of 50% H_2_SO_4_. The produced 2-acetolactate then underwent acid hydrolysis to form acetoin, which could be quantified using the Voges–Proskauer assay by measuring the absorbance of the red mixture at 535 nm [40]. The enzyme activity was represented as the amount of 2-acetolactate produced by 1 mg of total protein in 1 min.

### Gas chromatography (GC) detection of higher alcohols

Higher alcohols were quantified using an Agilent 6890 GC with flame ionization detector. A DB-FFAP capillary column (30 m × 0.32 mm × 0.25 μm; Agilent Technologies) was used to separate C5 and C4 alcohols with n-pentanol as the internal standard. For the analysis of higher alcohols in aqueous phase, the GC oven temperature was first held at 80 °C for 3 min, increased to 230 °C at 115 °C min^− 1^, and held for 1 min. The alcohols in organic phase were separated by first holding the oven temperature at 90 °C for 0.5 min followed by heating at a rate of 20 °C min^−1^ to 110 °C and holding for 0.5 min. The temperature was then increased to 235 °C at 120 °C min^−1^ and held for 2 min. Samples were injected at a split ratio of 1:50 and detected at 280 °C.

### High-performance liquid chromatography detection of amino acids

The concentrations of amino acids were measured using an Agilent 1290 Infinity™ II liquid chromatography system equipped with a Durashell C18(L) column (Bonna-Agela Technologies, Torrance, CA, USA) using the phenylisothiocyanate derivatization method [41]. A solution containing 0.1 M sodium acetate (pH 6.5) and acetonitrile in volumetric ratio of 99.3:0.7, and a solution containing 80% acetonitrile were used as mobile phases [42]. The derived amino acids were detected at 254 nm using a diode array detector.

## Supplementary information

**Additional file 1: Table S1.** σ^54^-dependent promoters characterized in this study. **Table S2.** List of primers. **Figure S1.** The growth curves of the parent strain and the ammonia-assimilation-pathway-deleted strain in defined media. **Figure S2.** The biofuel titer of the LM19 and LM10 with biofuel synthetic pathway in M9 medium with 40 g L^− 1^ yeast extract as the carbon and nitrogen sources. **Figure S3.** The fluorescence intensities for GFP expressed from different σ^54^-dependent promoters. **Figure S4.** Activities of AlsS in the biofuel synthetic pathway driven by different promoters. **Figure S5.** Biofuel production from pathway driven by **a***glnAp2*, **b***argTp*, **c***P*_L_lacO_1_, **d** J23100 and **e***rrnBp1* in batch fermentation. **Figure S6.** The percentage of theoretical yield (g of product per g of consumed raw material) for biofuel produced from pathway driven by different promoters. **Figure S7.** The regulatory cascade for nitrogen assimilation in *E. coli*. **Figure S8.** Precursors of the 20 amino acids and their value-added derivatives. **Figure S9.** Amino acid composition of different protein sources.

## Data Availability

All data generated or analyzed during this study are included in this published article.

## Abbreviations

RNAP: RNA polymerase
Eσ^70^: RNAP-σ^70^ holoenzyme
Eσ^54^: RNAP-σ^54^ holoenzyme
KIV: 2-Ketoisovalerate
KMV: 2-Keto-3-methyl-valerate
KIC: 2-Ketoisocaproate
NtrB: Nitrogen regulatory protein B
NtrC: Nitrogen regulatory protein C
GDH: Glutamate dehydrogenase
GS: Glutamine synthetase
GOGAT: Glutamate synthase
GFP: Green fluorescent protein
QS: Quorum sensing
AI-2: Autoinducer-2
BCAA: Branched-chain amino acid
