# Supplementary material for: Protein-based biorefining driven by nitrogen-responsive transcriptional machinery
Source: Biotechnol Biofuels. 2020 Feb 26;13:29. doi: 10.1186/s13068-020-1667-5 (PMC7045595; doi:10.1186/s13068-020-1667-5)
Supplement: Supplementary file 1 — Additional file 1: Table S1. σ54-dependent promoters characterized in this study. Table S2. List of primers. Figure S1. The growth curves of the parent strain and the ammonia-assimilation-pathway-deleted strain in defined media. Figure S2. The biofuel titer of the LM19 and LM10 with biofuel synthetic pathway in M9 medium with 40 g L− 1 yeast extract as the carbon and nitrogen sources. Figure S3. The fluorescence intensities for GFP expressed from different σ54-dependent promoters. Figure S4. Activities of AlsS in the biofuel synthetic pathway driven by different promoters. Figure S5. Biofuel production from pathway driven by aglnAp2, bargTp, cPLlacO1, d J23100 and errnBp1 in batch fermentation. Figure S6. The percentage of theoretical yield (g of product per g of consumed raw material) for biofuel produced from pathway driven by different promoters. Figure S7. The regulatory cascade for nitrogen assimilation in E. coli. Figure S8. Precursors of the 20 amino acids and their value-added derivatives. Figure S9. Amino acid composition of different protein sources. [file 13068_2020_1667_MOESM1_ESM.pdf]

Table S1 σ^{54}-dependent promoters characterized in this study

Promoters	Genes	Gene functions
glnHp2	*glnH*	periplasmic binding protein of L-glutamine ABC transporter
ddpXp	*ddpX*	D-alanyl-D-alanine dipeptidase
glnKp	*glnK*	nitrogen regulator PII
astCp2	*astC*	succinylornithine transaminase
pabBp6	*pabB*	aminodeoxychorismate synthase subunit I
patAp	*patA*	putrescine aminotransferase
puuPp	*puuP*	proton dependent putrescine transporter
rutAp	*rutA*	pyrimidine oxygenase
yhdWp	*yhdW*	substrate binding protein of a putative polar amino acid transporter
argTp	*argT*	periplasmic binding protein of a basic amino acid ABC transporter
nacp	*nac*	DNA-binding transcriptional dual regulator
glnAp2	*glnA*	glutamine synthetase

Table S2 List of primers

Primers	Sequence 5'-3'
Primers for pathway construction	
pLM-F	cctttcgtcttcacctcgagacattcacatcgtggtgcagc
pLM-R	ctcgaggtgaagacgaaagg
pLM1-F	acgcgtgctagaggcatc
pLM1-R	ttaacccccagtttcgatttatc
pLM6-F	agatttcgctttatcttttgaattcattaaagaggagaaagg
pLM6-R	caccatgttggtgcaatgacctcgaggtgaagacgaaag
pLM8-F	ataagacctgcatgaaagagcgaattcattaaagaggaga
pLM8-R	aattgccctgaaacagggcactcgaggtgaagacgaaagg
avta-F	aatcgaaactgggggttaaacgcgtaagaaggagatataccatg
avta-R	ttgatgcctctagcacgcgtttagtgactttcagcccagg
glnAp2-F	gccctttcgtcttcacctcgaggtcattgcaccaacatggtg
glnAp2-R	ctttctcctctttaatgaattcaaaaagataaagcgaaatctgtgccaac
glnAp2-F2	gtcattgcaccaacatggtg
glnAp2-R2	aaaaagataaagcgaaatctgtgc
rrnBp-F	cctttcgtcttcacctcgagcaagtgctgccagagggaac
rrnBp-R	ttctcctctttaatgaattccccggcggcgtgtttgccgt
J23100-M1-F	tcagtcctaggtacagtgctagcgaattcattaaagaggagaaaggtacaatgttgacaaaag
J23100-97-F	tcagtcctaggtacagtgctagcgaattcattaaagaggagaaaggtaccatgaaaatct
J23100-R	actgtacctaggactgagctagccgtcaactcgaggtgaagacgaaagggcctc
Primers for promoter selection	
pLMg1-F	acgcgtgctagaggcatc
pLMg1-R	tgtacctttctcctctttaa
gfp-F	cattaaagaggagaaaggtacaatggtgagcaagggcgag
gfp-R	gatgcctctagcacgcgtttacttgtacagctcgtccatg
argTp-F	cctttcgtcttcacctcgagtgccctgtttcagggcaatt
argTp-R	ttctcctctttaatgaattcggctctttcatgcaggtctt
astCp-F	cctttcgtcttcacctcgagatgtcaacgatggcgcaaaa
astCp-R	ttctcctctttaatgaattcaaatgtagattgcagggttc
ddpXp-F	cctttcgtcttcacctcgagcttcccctcttgcaccaaaac
ddpXp-R	tctcctctttaatgaattccgctttccgcttatgcagatc
glnHp-F	cctttcgtcttcacctcgagaatcactgtgttgagtgcac
glnHp-R	ttctcctctttaatgaattcattcacatatatgaaaaaat
glnKp-F	cctttcgtcttcacctcgagatagcgcaatatttcatcgt
glnKp-R	ttctcctctttaatgaattcaagaaggtattgcaagcggt

nacp-F	cctttcgtcttcacctcgagtgaaccatcgtggtgcatac
nacp-R	ttctcctctttaatgaattcacaaccagattgcaagatgc
pabBp-F	cctttcgtcttcacctcgagcacgattcgctcgccggagt
pabBp-R	tctcctctttaatgaattccgggtagcatgaaataattag
patAp-F	cctttcgtcttcacctcgagcgcaccatgttgtgcggctg
patAp-R	ttctcctctttaatgaattcatttaagtattgcagggatt
puuPp-F	cctttcgtcttcacctcgagtgcagtttgagcgcctcatc
puuPp-R	ttctcctctttaatgaattcaacggaagcccgttgccgcc
rutAp-F	cctttcgtcttcacctcgagtgcactctcatcgcgcacag
rutAp-R	ttctcctctttaatgaattcgtattggcttgtttgcaaag
yhdWp-F	cctttcgtcttcacctcgagtgcgccaaaatgtggcgcat
yhdWp-R	ttctcctctttaatgaattctcctcttataagcaaaagta
glnAp2g-F	cctttcgtcttcacctcgagacattcacatcgtggtgcag
glnAp2g-R	ttctcctctttaatgaattcaaaaagataaagcgaaatct

To clone pLM1, primers pLM1-F and pLM1-R were used to amplify $P_L$lacO_1: *alsS-ilvC-ilvD* from pSA69, primers avta-F and avta-R were used to amplify *avtA* from the *E. coli* genomic DNA, the PCR products were assembled by Gibson Assembly.

To clone pLM2, primers rrnBp-F and rrnBp-R were used to amplify *rrnBp1* from the *E. coli* genomic DNA, primers pLM-F and pLM-R were used to amplify *alsS-ilvC-ilvD-avtA* from pLM1, the PCR products were assembled by Gibson Assembly.

To clone pLM3, primers rrnBp-F and rrnBp-R were used to amplify *rrnBp1* from the *E. coli* genomic DNA, primers pLM-F and pLM-R were used to amplify *leuDH-kivD-yqhD; lacI* from pYX97, the PCR products were assembled by Gibson Assembly.

To clone pLM4, primers J23100-M1-F and J23100-R were used to amplify J23100: *alsS-ilvC-ilvD-avtA* from pLM1, the PCR product was assembled by Gibson Assembly.

To clone pLM5, primers J23100-97-F and J23100-R were used to amplify J23100: *leuDH-kivD-yqhD; lacI* from pYX97, the PCR product was assembled by Gibson Assembly.

To clone pLM6, primers pLM6-F and pLM6-R were used to amplify *glnAp2* from the *E. coli* genomic DNA, primers pLM-F and pLM-R were used to amplify *alsS-ilvC-ilvD-avtA* from pLM1, the PCR products were assembled by Gibson Assembly.

To clone pLM7, primers pLM6-F and pLM6-R were used to amplify *glnAp2* from the *E. coli* genomic DNA, primers pLM-F and pLM-R were used to amplify *leuDH-kivD-yqhD; lacI* from pYX97, the PCR products were assembled by Gibson Assembly.

To clone pLM8, primers pLM8-F and pLM8-R were used to amplify *argTp* from the *E. coli* genomic DNA, primers pLM-F and pLM-R were used to amplify *alsS-ilvC-ilvD-avtA* from pLM1, the PCR products were assembled by Gibson Assembly.

To clone pLM9, primers pLM8-F and pLM8-R were used to amplify *argTp* from the *E. coli* genomic DNA, primers pLM-F and pLM-R were used to amplify *leuDH-kivD-yqhD; lacI* from pYX97, the PCR products were assembled by Gibson Assembly.

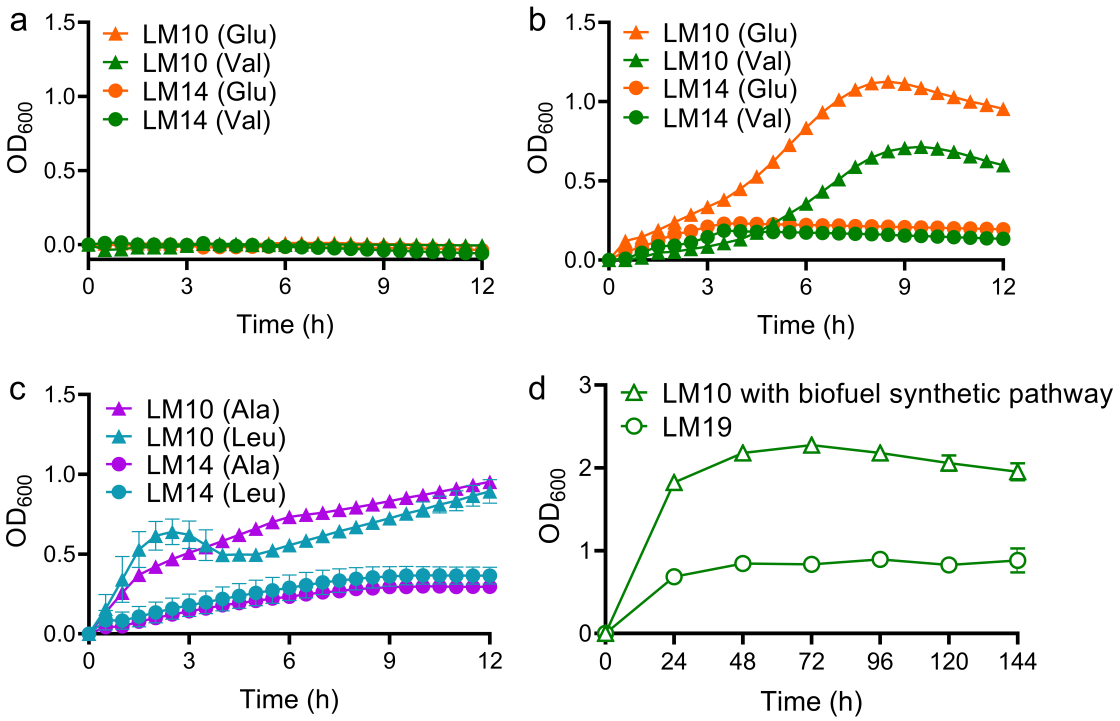

Figure S1 The growth curves of the parent strain and the ammonia-assimilation-pathway-deleted strain in defined media. **a** The growth curve of LM10 and LM14 in M9 medium with 15 mM valine (Val) or 15 mM glutamine (Glu) as the sole carbon and nitrogen sources. **b** The growth curve of LM10 and LM14 in M9 medium with 0.4% glucose as the carbon source and 15 mM Val or 15 mM Glu as the nitrogen source. **c** the growth curve of LM10 and LM14 in M9 medium with 0.5% yeast extract as the carbon source and 15 mM alanine (Ala) or 15 mM leucine (Leu) as the nitrogen source. **d** The growth curve of LM10 with isobutanol producing pathway and LM19 in M9 medium with 0.4% glucose as the carbon source and 15 mM glutamine as the nitrogen source.

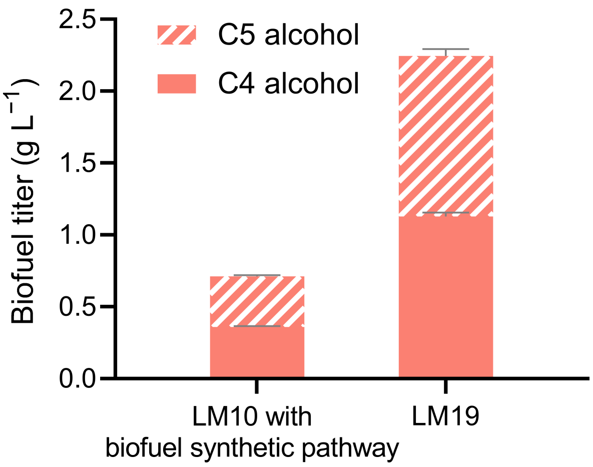

Figure S2 The biofuel titer for LM19 and LM10 with biofuel synthetic pathway in M9 medium with 40 g L^{-1} yeast extract as the carbon and nitrogen sources.

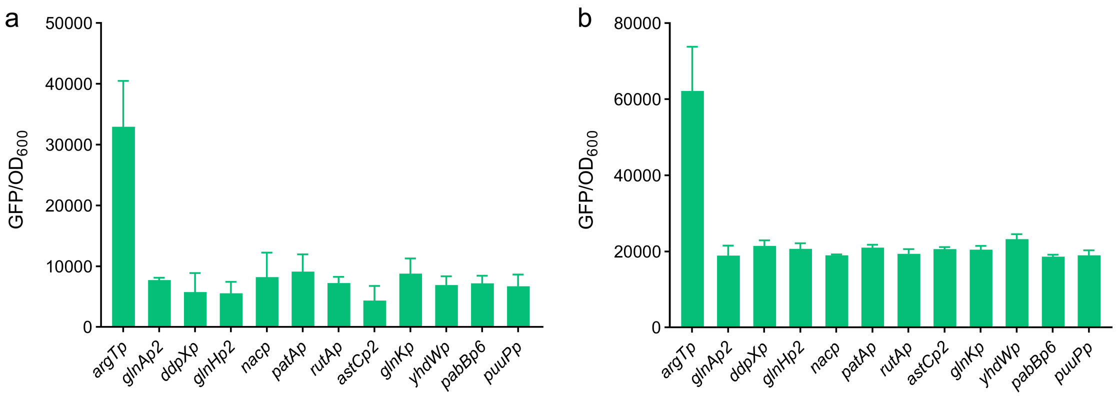

Figure S3 The fluorescence intensities for GFP expressed from different σ^{54}-dependent promoters. **a** Fluorescence intensities for cells in the early stationary phase. **b** Fluorescence intensities for cells in the late stationary phase. All the strains were cultured in a 96 deep-well (2 mL) plate and sampled at defined time points for measurement of fluorescence intensity. Values and error bars represent the mean and the s.d. (n = 3).

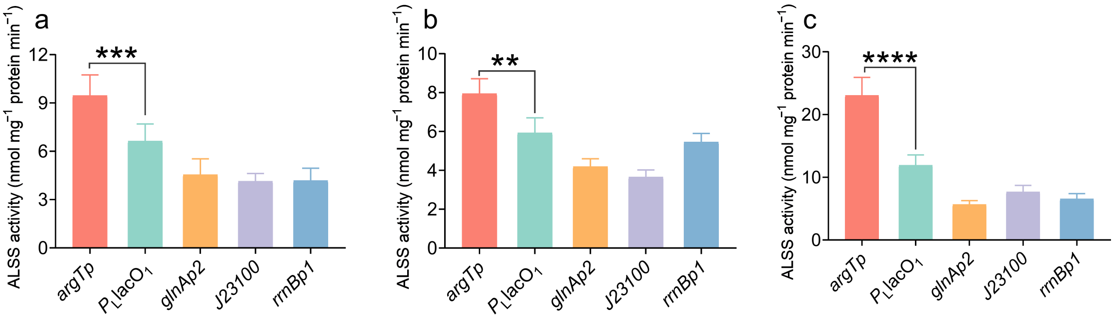

Figure S4 Activities of AlsS in the biofuel synthetic pathway driven by different promoters. **a** AlsS activities under optimal condition; **b** AlsS activities under osmotic stress (400 mM NaCl); **c** AlsS activities under acid stress (pH 5.0). Values and error bars represent the mean and the s.d. (n = 3). $**P < 0.01$, $***P < 0.001$, $****P < 0.0001$ as determined by two-tailed *t* test.

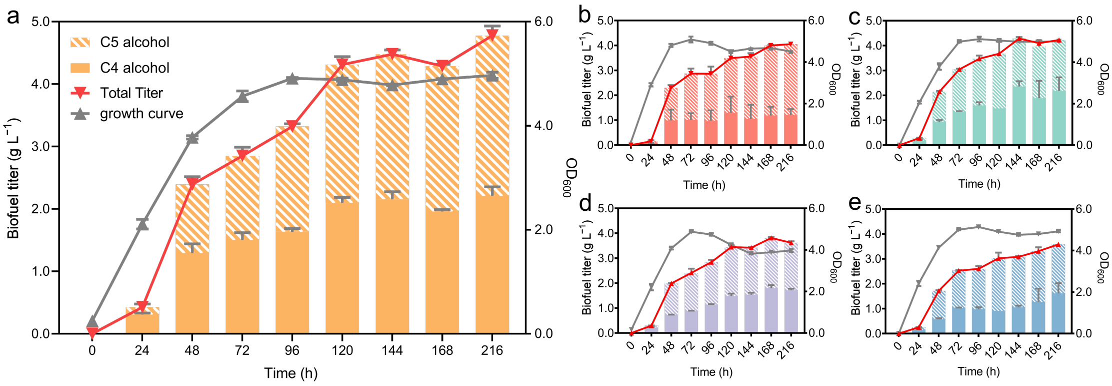

Figure S5 Biofuel production from pathway driven by **a** *glnAp2*, **b** *argTp*, **c** $P_L$lacO_1, **d** J23100 and **e** *rrnBp1* in batch fermentation.

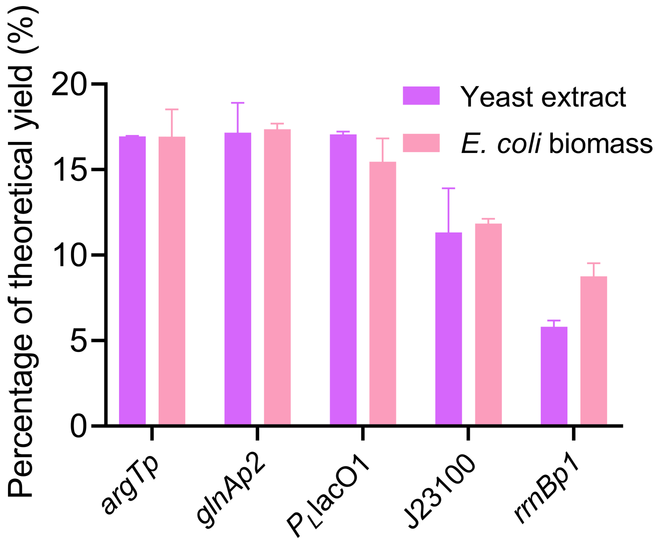

Figure S6 The percentage of theoretical yield [1] (g of product per g of consumed raw material) for biofuel produced from pathway driven by different promoters. Yeast extract and *E. coil* protein biomass were used as the feedstock.

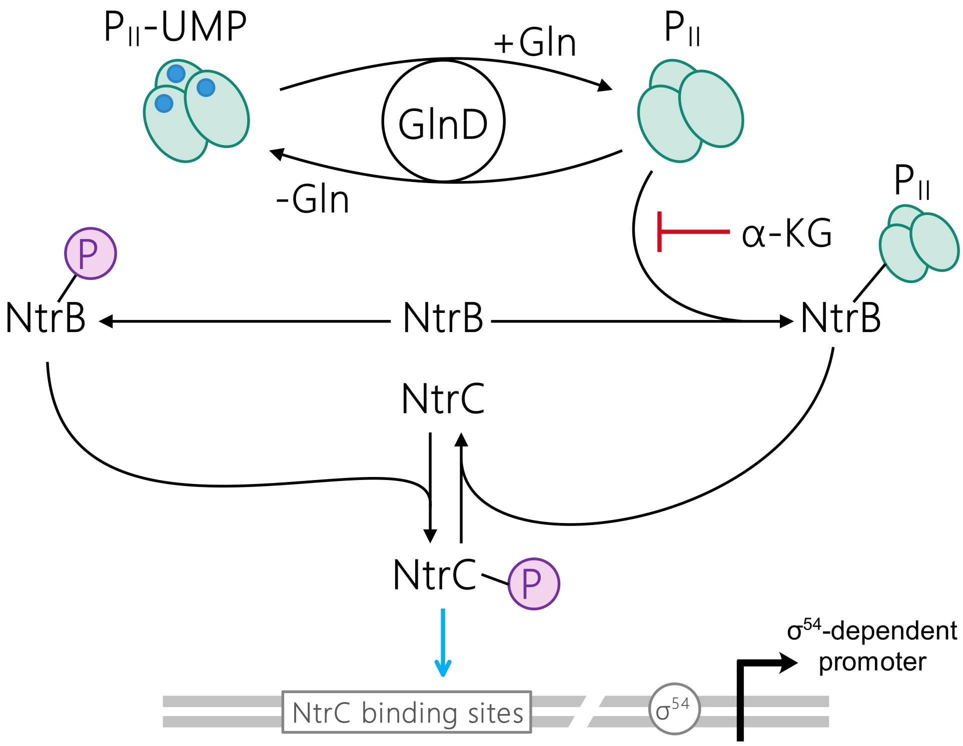

Figure S7 The regulatory cascade for nitrogen assimilation in *E. coli*. The nitrogen status is sensed by the GlnD and the P_{II} system. A low nitrogen availability promotes GlnD to uridylylate P_{II} protein (P_{II}-UMP) and inhibit its ability to bind NtrB, allowing free NtrB to undergo phosphorylation and subsequently transfer its phosphoryl group to NtrC. The phosphorylated NtrC (NtrC-P) then activates *glnAp2* and other targeted promoters [2]. The GlnD responds to intracellular glutamine and the PII is regulated by 2-oxoglutarate [3]. Accumulation of glutamine (Gln) promotes GlnD to deuridylylate PII-UMP and leads to the dephosphorylation of NtrC-P, which subsequently suppresses the NtrC-mediated transcription. Excessive α-ketoglutarate (α-KG) would bind with PII and promote the release of free NtrB. The increased level of NtrB available for phosphorylation would subsequently increase the level of NtrC-P and activate the targeted transcriptions.

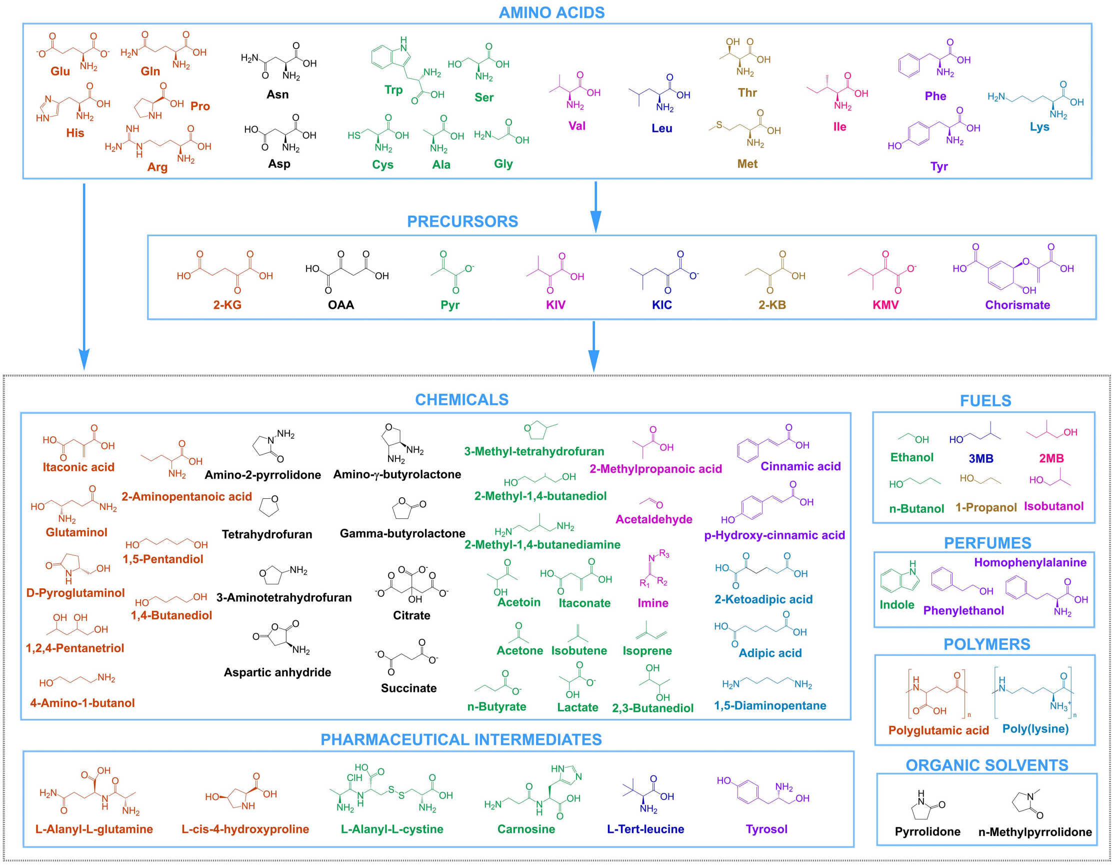

Figure S8 Precursors of the 20 amino acids and their value-added derivatives. The amino acids and their corresponding precursors and derivatives are of the same color. 2-KG: 2-ketoglutarate, OAA: oxaloacetic acid, PYR: pyruvic acid, KIV: 2-ketoisovaleric acid, KIC: 2-ketoisocaproic acid, 2-KB: α-ketobutyrate, KMV: 2-ketomethylvaleric acid. The derivatives include fuels (e.g. ethanol [4], 2-methyl-1-butanal (2MB), 3-methyl-1-butanol (3MB), isobutanol [1], n-butanol [5] and 1-propanol [6]), perfumes (e.g., indole [7], phenylethanol [8] and homophenylalanine [9]), polymers (e.g., polyglutamic acid [10] and polylysine [11]), organic solvents (e.g., pyrrolidone [12] and n-methylpyrrolidone [13]), pharmaceutical intermediates (e.g., L-alanyl-L-glutamine [14], L-cis-4-hydroxyproline, L-alanyl-L-cystine, carnosine [15], L-tert-leucine [16], and tyrosol [18]), and other chemicals (e.g., itaconic acid [19], 1,5-diaminopentane [20], 1,5-pentandiol [21], 4-amino-1-butanol [22], D-pyroglutaminol [21], 2-aminopentanoic acid [23], 1,2,4-pentanetriol [24], glutaminol [25], amino-γ-butyrolactone [27], amino-2-pyrrolidone [12], tetrahydrofuran [1], γ-butyrolactone [21], 3-aminotetrahydrofuran [21], aspartic anhydride [27], citrate [28], 1,4-butanediol [29], succinate [30], 2,3-butanediol [31], isoprene [32], n-butyrate [33], acetoin [31], acetone

[34], itaconate [35], isobutene [36], lactate [33], 3-methyl-tetrahydrofuran [37], 2-methyl-1,4-butanediol [38], 2-methyl-1,4-butanediamine [39], 2-methylpropanoic acid [40], acetaldehyde [41], imine [42], cinnamic acid [43], p-hydroxy-cinnamic acid [44], 2-ketoadipic acid [42] and adipic acid [45]).

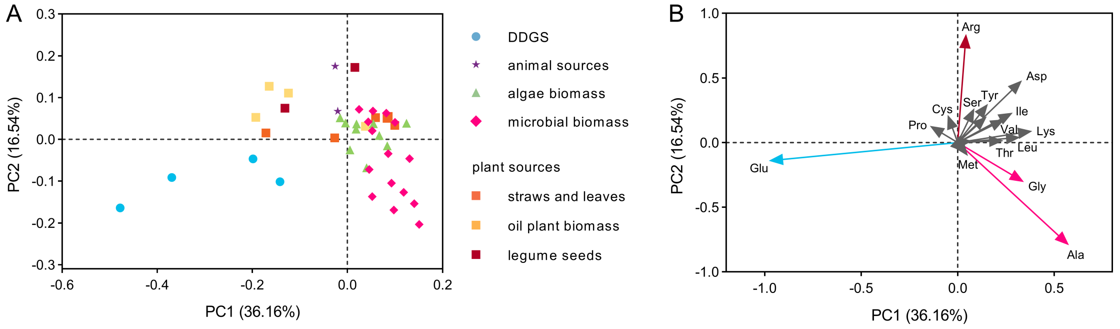

Figure S9 Amino acid composition of different protein sources. **a** Principle component analysis (PCA) of the amino acid composition of different raw materials, the proportion of variance accounted for by the two axes are presented in the parenthesis. **b** The distribution of individual amino acids in the corresponding PCA space. When projected onto the vectors, protein sources with projections located in the positive direction of a vector have higher abundance of the corresponding amino acid than that of other materials. For instance, the distillers dried grains with solubles (DDGS) is rich in glutamic acid and the bacterial biomass has a relatively high abundance of alanine and glycine. The DDGS used for analysis are sorghum DDGS [46], maize DDGS, wheat DDGS, and sugarbeet vinasse [47]; the animal sources of proteins are poultry feathers [47] and eggs [48]; the algae biomass include *Gracilaria* sp.[49], *Dromogomphus armatus* [50], *Chlorella* sp., *Spirulina* sp. [47], *Aphanizomenon* sp., *Chlorella vulgaris*, *Dunaliella bardawil*, *Scenedesmus obliquus*, *Arthrospira maxima*, and *pirulina platensis* [48]; the microbial biomass include *Saccharomyces cerevisiae*, *Kluyveromyces marxianus*, *Saccharomyces uvarum*, *Saccharomycodes ludwigii* [51], *Brevibacterium methylicum* [52], *Bacillus subtilis* [52], *Rhodocyclus gelatinosus* [53], *Rhodopseudomonas capsulate* [53], *Corynebacterium glutamicum* [54], *Staphylococcus aureus* [55], *Lactobacillus acidophilus*, *Lactobacillus bulgaricus*, *Lactobacillus casei*, *Lactobacillus plantarum*, and *Streptococcus thermophilus* [56]; the straws and leaves protein sources are sugarbeet leaves [57], sugarcane leaves [58], cassava leaves [59], ryegrass meal [60], wheat straw and alfalfa hay [47]; the oil plant biomass are from jatropha meal [61], sunflower seed meal, rapeseed, and palmoil meal [47]; the legume seed biomass are soybeen [62] and carob germ [63].

References

1. Huo YX, Cho KM, Rivera JG, Monte E, Shen CR, Yan Y, Liao JC. Conversion of proteins into biofuels by engineering nitrogen flux. Nat Biotechnol. 2011;29:346-351.
2. van Heeswijk WC, Westerhoff HV, Boogerd FC. Nitrogen assimilation in *Escherichia coli*: putting molecular data into a systems perspective. Microbiol Mol Biol Rev. 2013;77:628.
3. Huergo LF, Chandra G, Merrick M. P_{II} signal transduction proteins: nitrogen regulation and beyond. FEMS Microbiol Rev. 2013;37:251-283.
4. Yang M, Li X, Bu C, Wang H, Shi G, Yang X, Hu Y, Wang X. Pyruvate decarboxylase and alcohol dehydrogenase overexpression in *Escherichia coli* resulted in high ethanol production and rewired metabolic enzyme networks. World J Microbiol Biotechnol. 2014;30:2871-2883.
5. Si T, Luo Y, Xiao H, Zhao H. Utilizing an endogenous pathway for 1-butanol production in *Saccharomyces cerevisiae*. Metab Eng. 2014;22:60-68.
6. Shen CR, Liao JC. Synergy as design principle for metabolic engineering of 1-propanol production in *Escherichia coli*. Metab Eng. 2013;17:12-22.
7. Sasaki-Imamura T, Yoshida Y, Suwabe K, Yoshimura F, Kato H. Molecular basis of indole production catalyzed by tryptophanase in the genus *Prevotella*. FEMS Microbiol Lett. 2011;322:51-59.
8. Pan X, Qi H, Mu L, Wen J, Jia X. Comparative metabolomic-based metabolic mechanism hypothesis for microbial mixed cultures utilizing cane molasses wastewater for higher 2-phenylethanol production. J Agric Food Chem. 2014;62:9927-9935.
9. Koketsu K, Mitsuhashi S, Tabata K. Identification of homophenylalanine biosynthetic genes from the cyanobacterium *Nostoc punctiforme* PCC73102 and application to its microbial production by *Escherichia coli*. Appl Environ Microbiol. 2013;79:2201-2208.
10. Hao J, Shang L, Yoon SH, Lee SY, Yu Z. Optimal production of poly-γ-glutamic acid by metabolically engineered *Escherichia coli*. Biotechnol Lett. 2006;28:1241-1246.
11. Oppermann-Sanio FB, Steinbüchel A. Occurrence, functions and biosynthesis of polyamides in microorganisms and biotechnological production. Naturwissenschaften. 2002;89:11-22.
12. Zhang J, Kao E, Wang G, Baidoo EEK, Chen M, Keasling JD. Metabolic engineering of *Escherichia coli* for the biosynthesis of 2-pyrrolidone. Metab Eng Commun. 2016;3:1-7.
13. Lammens TM, Franssen MCR, Scott EL, Sanders JPM. Synthesis of biobased N-methylpyrrolidone by one-pot cyclization and methylation of γ-aminobutyric acid. Green Chem. 2010;12:1430-1436.
14. Tabata K, Hashimoto S-i. Fermentative production of L-alanyl-L-glutamine by a metabolically engineered *Escherichia coli* strain expressing L-amino acid α-ligase. Appl Environ Microbiol. 2007;73:: 6378–6385.
15. Grayson I, Kessler C. Modern applications of amino acids and dipeptides. Chim Oggi-Chem Today. 2015;33:46-51.
16. Liu W, Ma H, Luo J, Shen W, Xu X, Li S, Hu Y, Huang H. Efficient synthesis of l-tert-leucine through reductive amination using leucine dehydrogenase and formate dehydrogenase coexpressed in recombinant *E. coli*. Biochem Eng J. 2014;91:204-209.
17. Lo HH, Hsu SK, Lin WD, Chan NL, Hsu WH. Asymmetrical synthesis of L-homophenylalanine using engineered *Escherichia coli* aspartate aminotransferase. Biotechnol Prog. 2005;21:411-415.
18. Chung D, Kim SY, Ahn J-H. Production of three phenylethanoids, tyrosol, hydroxytyrosol, and

salidroside, using plant genes expressing in *Escherichia coli*. Sci Rep. 2017;7:2578.
19. Shusuke O, Taejun C, Ken H, Yuji A, Yasutomo T, Tetsuya T, Hitomi O. Production of itaconic acid using metabolically engineered *Escherichia coli*. J Gen Appl Microbiol. 2014;60:191.
20. Buschke N, Becker J, Schäfer R, Kiefer P, Biedendieck R, Wittmann C. Systems metabolic engineering of xylose-utilizing *Corynebacterium glutamicum* for production of 1, 5-diaminopentane. Biotechnol J. 2013;8:557-570.
21. Werpy T, Petersen G: Top value added chemicals from biomass: volume I—results of screening for potential candidates from sugars and synthesis gas. Golden, CO (US): National Renewable Energy Lab; 2004.
22. Isikgor FH, Becer CR. Lignocellulosic biomass: a sustainable platform for the production of bio-based chemicals and polymers. Polymer Chemistry. 2015;6:4497-4559.
23. Atsumi S, Hanai T, Liao JC. Non-fermentative pathways for synthesis of branched-chain higher alcohols as biofuels. Nature. 2008;451:86.
24. Cao Y, Niu W, Guo J, Xian M, Liu H. Biotechnological production of 1,2,4-butanetriol: an efficient process to synthesize energetic material precursor from renewable biomass. Sci Rep. 2015;5:18149.
25. Giovannercole F, Pennacchietti E, De Biase D. L-Glutamate decarboxylase from bacteria. Methods Enzymol. 2017:15.
26. Cordes T, Michelucci A, Hiller K. Itaconic acid: the surprising role of an industrial compound as a mammalian antimicrobial metabolite. Annu Rev Nutr. 2015;35:451-473.
27. Piao X, Wang L, Lin B, Chen H, Liu W, Tao Y. Metabolic engineering of *Escherichia coli* for production of L-aspartate and its derivative β-alanine with high stoichiometric yield. Metab Eng. 2019;54:244-254.
28. Kobayashi K, Hattori T, Honda Y, Kirimura K. Oxalic acid production by citric acid-producing *Aspergillus niger* overexpressing the oxaloacetate hydrolase gene *oahA*. J Ind Microbiol Biotechnol. 2014;41:749-756.
29. Yim H, Haselbeck R, Niu W, Pujol-Baxley C, Burgard A, Boldt J, Khandurina J, Trawick JD, Osterhout RE, Stephen R, et al. Metabolic engineering of *Escherichia coli* for direct production of 1,4-butanediol. Nat Chem Biol. 2011;7:445.
30. Bodor Z, Fazakas A, Kovacs E, Lanyi S, Albert B. Systems biology and metabolic engineering for obtaining *E. coli* mutants capable to produce succinate from renewable resources. Rom Biotech lett. 2014;19:9633-9644.
31. Nielsen DR, Yoon SH, Yuan CJ, Prather KLJ. Metabolic engineering of acetoin and *meso*-2, 3-butanediol biosynthesis in *E. coli*. Biotechnol J. 2010;5:274-284.
32. Bredow S, Doneske S, Li M, Zhou H, Monticello DJ, Campbell P: Microorganisms and processes for the conversion of glycerol to isoprene. In US Patent Application 14/598,485; 2015.
33. Detman A, Mielecki D, Chojnacka A, Salamon A, Błaszczyk MK, Sikora A. Cell factories converting lactate and acetate to butyrate: *Clostridium butyricum* and microbial communities from dark fermentation bioreactors. Microb Cell Fact. 2019;18:36-36.
34. Zhou J, Zhang H, Zhang Y, Li Y, Ma Y. Designing and creating a modularized synthetic pathway in cyanobacterium *Synechocystis* enables production of acetone from carbon dioxide. Metab Eng. 2012;14:394-400.
35. Vuoristo KS, E. MA, Stijn vL, Enrico O, Gerrit E, M. SJP, A. WR. Heterologous expression of *Mus musculus* immunoresponsive gene 1 (*irg1*) in *Escherichia coli* results in itaconate

production. Front Microbiol. 2015;6:849.
36. Koepke M, Jensen RO: Genetically engineered bacterium for the production of isobutylene. In US Patent Application 15/922,451; 2018.
37. Klement T, Milker S, Jäger G, Grande PM, de María PD, Büchs J. Biomass pretreatment affects *Ustilago maydis* in producing itaconic acid. Microb Cell Fact. 2012;11:43.
38. Jang YS, Kim B, Shin JH, Choi YJ, Choi S, Song CW, Lee J, Park HG, Lee SY. Bio-based production of C2–C6 platform chemicals. Biotechnol Bioeng. 2012;109:2437-2459.
39. Kuenz A, Krull S. Biotechnological production of itaconic acid—things you have to know. Appl Microbiol Biotechnol. 2018;102:3901-3914.
40. Gutsche KA, Tran TBT, Vogel RF. Production of volatile compounds by *Lactobacillus sakei* from branched chain α-keto acids. Food Microbiol. 2012;29:224-228.
41. Balagurunathan B, Tan L, Zhao H. Metabolic engineering of *Escherichia coli* for acetaldehyde overproduction using pyruvate decarboxylase from *Zymomonas mobilis*. Enzyme Microb Technol. 2018;109:58-65.
42. Zabriskie TM, Jackson MD. Lysine biosynthesis and metabolism in fungi. Nat Prod Rep. 2000;17:85-97.
43. Gottardi M, Knudsen JD, Prado L, Oreb M, Branduardi P, Boles E. De novo biosynthesis of *trans*-cinnamic acid derivatives in *Saccharomyces cerevisiae*. Appl Microbiol Biotechnol. 2017;101:4883-4893.
44. Vargas-Tah A, Martínez L, Hernández-Chávez G, Rocha M, Martínez A, Bolívar F, Gosset G. Production of cinnamic and *p*-hydroxycinnamic acid from sugar mixtures with engineered *Escherichia coli*. Microb Cell Fact. 2015;14:1-12.
45. Zhao M, Huang D, Zhang X, Koffas MAG, Deng Y. Metabolic engineering of *Escherichia coli* for producing adipic acid through the reverse adipate-degradation pathway. Metab Eng. 2018;47:254-262.
46. Urriola PE, Hoehler D, Pedersen C, Stein HH, Shurson GC. Amino acid digestibility of distillers dried grains with solubles, produced from sorghum, a sorghum-corn blend, and corn fed to growing pigs. J Anim Sci. 2009;87:2574-2580.
47. Deike V, Mol JD: Raw material compendium. 2 ed edn: Brussels Novus International Inc.; 1996.
48. Becker EW. Micro-algae as a source of protein. Biotechnol Adv. 2007;25:207-210.
49. Wen X, Peng C, Zhou H, Lin Z, Lin G, Chen S, Li P. Nutritional composition and assessment of *Gracilaria lemaneiformis* bory. J Integr Plant Biol. 2006;48:1047-1053.
50. Cheban L, Malischuk I, Marchenko M. Cultivating *Desmodesmus armatus* (Chod.) Hegew. in recirculating aquaculture systems (RAS) waste water. Arch Pol Fish. 2015;23:155-162.
51. Martini AEV, Miller MW, Martini A. Amino acid composition of whole cells of different yeasts. J Agric Food Chem. 1979;27:982-984.
52. Mosin O, Ignatov I, Skladnev D, Shvets V. Studying physiological, morphological, and cytological alterations in prokaryotic and eukaryotic cells in heavy water. Russ J Biol Res. 2015;5:143-172.
53. Ponsano EHG, Lacava PM, Pinto MF. Chemical composition of *Rhodocyclus gelatinosus* biomass produced in poultry slaughterhouse wastewater. Braz Arch Biol Techn. 2003;46:143-147.
54. Nishio Y, Nakamura Y, Kawarabayasi Y, Usuda Y, Kimura E, Sugimoto S, Matsui K, Yamagishi A, Kikuchi H, Ikeo K. Comparative complete genome sequence analysis of the amino acid

replacements responsible for the thermostability of *Corynebacterium efficiens*. Genome Res. 2003;13:1572-1579.

55. Okayasu T, Ikeda M, Akimoto K, Sorimachi K. The amino acid composition of mammalian and bacterial cells. Amino Acids. 1997;13:379-391.

56. Erdman MD, Bergen WG, Reddy CA. Amino acid profiles and presumptive nutritional assessment of single-cell protein from certain lactobacilli. Appl Environ Microbiol. 1977;33:901-905.

57. Merodio C, Sabater B. Preparation and properties of a white protein fraction in high yield from sugar beet (*Beta vulgaris* L) leaves. J Sci Food Agric. 1988;44:237-243.

58. Deepchand K, Baguant J. Utilisation of cane tops and leaves for energy and food — an integrated system. Biomass. 1985;7:215-223.

59. Fasuyi AO, Aletor VA. Varietal composition and functional properties of cassava (*Manihot esculenta*, *Crantz*) leaf meal and leaf protein concentrates. Pakistan J Nutr. 2005;4:43-49.

60. Eppendorfer WH. Amino acid composition and nutritional value of italian ryegrass, red clover and lucerne as influenced by application and content of nitrogen. J Sci Food Agric. 1977;28:607-614.

61. Martínez-Herrera J, Siddhuraju P, Francis G, Dávila-Ortíz G, Becker K. Chemical composition, toxic/antimetabolic constituents, and effects of different treatments on their levels, in four provenances of *Jatropha curcas* L. from Mexico. Food Chem. 2006:80-89.

62. Song X, Zang X, Zhang X. Production of high docosahexaenoic acid by *Schizochytrium* sp. using low-cost raw materials from food industry. J Oleo Sci. 2015;64:197-204.

63. Parrado J, Bautista J, Romero EJ, Garcia-Martinez AM, Friaza V, Tejada M. Production of a carob enzymatic extract: potential use as a biofertilizer. Bioresour Technol. 2008;99:2312-2318.
